# CD83^+^CCR7^+^ NK cells induced by interleukin 18 by dendritic cells promote experimental autoimmune uveitis

**DOI:** 10.1111/jcmm.14081

**Published:** 2018-12-08

**Authors:** Qiang Fu, Xuejing Man, Xin Wang, Nannan Song, Yuanbin Li, Jiangnan Xue, Yufei Sun, Wei Lin

**Affiliations:** ^1^ Department of Immunology Binzhou Medical University Yantai China; ^2^ Department of Ophthalmology Yuhuangding Hospital Yantai China; ^3^ Department of Clinical Laboratory Qilu Hospital of Shandong University Jinan China; ^4^ Institute of Basic medicine Shandong Academy of medical Sciences Jinan China

**Keywords:** autoimmune uveitis, dendritic cells, IL‐18, natural killer cells

## Abstract

Natural killer (NK) cells have been reported to play a pathological role in autoimmune uveitis. However, the mechanisms regarding NK cells in uveitis and factors that affect NK‐cell activation in this condition remain unclear. Here, we report that the number of CD3^‐^NK1.1^+^CD83^+^CCR7^+^ cells is increased in the inflamed eyes within a mouse model of experimental autoimmune uveitis (EAU), and these cells express elevated levels of NKG2D, CD69 and IFN‐γ. Adoptively transferring CD83^+^CCR7^+^NK cells aggravates EAU symptoms and increases the number of CD4^+^IFN‐γ^+^T cells and dendritic cells (DCs) within the eye. These CD83^+^CCR7^+^NK cells then promote the maturation of DCs and IFN‐γ expression within T cells as demonstrated in vitro. Furthermore, IL‐18, as primarily secreted by DCs in the eyes, is detected to induce CD83^+^CCR7^+^NK cells. In EAU mice, anti‐IL‐18R antibody treatment also decreases retinal tissue damage, as well as the number of infiltrating CD83^+^CCR7^+^NK cells, T cells and DCs in the inflamed eyes and spleens of EAU mice. These results suggest that CD83^+^CCR7^+^NK cells, as induced by IL‐18 that primarily secreted by DCs, play a critical pathological role in EAU. Anti‐IL‐18R antibody might serve as a potential therapeutic agent for uveitis through its capacity to inhibit CD83^+^CCR7^+^NK cells infiltration.

## 1 INTRODUCTION

Uveitis, an inflammatory disease involving the uvea, retina, retinal vessels and/or vitreous body, can result in visual impairment and blindness.^1^ This condition encompasses a variety of intraocular inflammations which can affect the anterior, middle, or posterior regions of the eye.^1^, ^2^ If uveitis is localized to the posterior region, it can affect photoreceptor cells and will likely lead to a visual disability.^1^, ^2^ A disorder of the immune system represents an essential pathogenesis for autoimmunity uveitis. In specific, the large number of lymphocytes, including mature dendritic cells (DCs), T cells and natural killer (NK) cells infiltrating the eye may be a critical factor which drives this disorder of the immune system to result in tissue damage.^1^, ^2^, ^3^, ^4^, ^5^, ^6^ However, the underlying mechanisms of uveitis remain unclear.

Recently, NK cells have been reported to play a role in experimental autoimmune uveitis (EAU).^3^, ^4^, ^6^, ^7^, ^8^ NK cells, which are a component of the innate immune system, represent the first line of defence against infections and play a regulatory role in autoimmune diseases by regulating the secretion of cytokines or by interacting with other cells.^9^, ^10^, ^11^, ^12^, ^13^, ^14^, ^15^ As activated NK cells produce a significant increase in the expression of IFN‐γ in Behcet’s disease, a type of autoimmune uveitis,^4^, ^6^ any prevention in the secretion of IFN‐γ from NK cells would have the potential to alleviate uveitis. An alteration in the status of NK cells may also contribute to the recovery of Behcet’s disease through suppression of Th1 responses,^4^, ^6^ which represents a pathogenic factor for uveitis.^6^, ^16^, ^17^, ^18^, ^19^ Depleting of NK cells would alleviate the symptom of EAU.^8^ Thus, the status of NK cells plays a critical role with regard to the development of autoimmune uveitis. Previous findings from our laboratory have revealed that EAU increased the number of CD3^‐^NK1.1^+^ NK cells during its inflammatory stages within the eyes and spleens.^3^ Moreover, these cells expressed increased levels of CD83 and secreted elevated levels of IFN‐γ.^3^ However, the role of such CD83^+^NK cells in EAU remains unknown and the specific factors affecting activation of NK cells in uveitis are unclear.

Interleukin‐18 is a pro‐inflammatory cytokine and is originally defined as an IFN‐γ‐inducing‐factor.^20^ It has been reported that IL‐18 promotes differentiation of the NK cell specifically, CD56^+^/CD83^+^/CCR7^+^ cells, as well as the migration of NK cells.^21^ However, whether IL‐18 promotes NK cell activation in uveitis is unknown. IL‐18 is constitutively produced by haematopoietic cells, such as DCs, macrophages and neutrophils,^22^, ^23^, ^24^ as well as in non‐hematopoietic cells, such as microglial and epithelial cells.^25^ IL‐18 involves binding with its receptor, consisting of IL‐18R1 and IL‐18R accessory protein in a heterodimeric receptor complex, to initiate signal transduction by the myeloid differentiation primary response protein 88 (MyD88). The nuclear factor (NF) kappa‐light‐chain‐enhancer of activated B cells (κB) and mitogen‐activated protein kinase pathways (MAPK pathways) are then activated, which promote IFN‐γ transcription and stabilization of IFNG mRNA.^26^, ^27^, ^28^ Recently, the role of IL‐18 in uveitis has attracted considerable attention as it has been shown in both clinical and EAU models, that IL‐18 shows enhanced expression during the inflammatory phase of the disease,^29^, ^30^, ^31^, ^32^, ^33^, ^34^ and polymorphism of the IL‐18 gene is closely related to the occurrence of uveitis.^35^, ^36^ Thus, these reports indicate that IL‐18 might participate in the inflammation of EAU. However, the mechanisms of IL‐18 in uveitis remain unknown.

In this report, we investigated the status of NK cells and their role in uveitis, and tested the capacity for IL‐18 to influence NK cell status in uveitis. And then, the possible cell subsets involved in secreting IL‐18 in uveitis were measured. In addition, we examined whether blocking of IL‐18 receptor would alleviate the development of uveitis and affect the status of CD83^+^CCR7^+^NK cells, DCs and T cells. The increases in IL‐18 observed in EAU might represent an important factor which promotes CD83^+^CCR7^+^ NK cell activation, thereby participating in the development of uveitis.

## 2 MATERIALS AND METHODS

### 2.1 Experimental autoimmune uveitis

Pathogen‐free female C57BL/6 (B6) mice (6‐8 weeks of age) were purchased from the Peking Vital River Laboratory Animal Ltd., Beijing, China and were maintained under pathogen‐free conditions, and were performed in compliance with guidelines of the care and use of laboratory animals were approved by the ethics committee of Shandong Academy of Medical Sciences (Jinan, China). Mice were immunized via subcutaneous injections at six locations (footpads, tail base, posterior neck and bilateral flanks) with 350 μg human interphotoreceptor retinoid‐binding protein peptide (IRBP) 1‐20 (ChinaPeptides Co., Ltd., Shanghai, China) emulsified in complete Freund’s adjuvant (Sigma, St. Louis, MO, USA). Concurrently, a single dose of 500 ng pertussis toxin (PTX; Enzo Life Sciences, Farmingdale, NY, USA) was injected intraperitoneally.^37^, ^38^ After immunization, the mice were examined by histopathology and clinical examination with a Genesis‐D camera (Kowa Company Ltd., Hamamatsn City, Japan). The clinical scores and histopathologic scores were showed in Tables S1 and S2, according to the previous description.^37^, ^38^, ^39^, ^40^

### 2.2 CD83^+^CCR7^+^ NK or CD83^‐^CCR7^‐^ NK cell adoptive transfers

CD3^‐^NK1.1^+^CD83^+^CCR7^+^ NK or CD3^‐^NK1.1^+^CD83^‐^CCR7^‐^ NK cells were sorted from EAU mice with use of a flow sorting instrument (BD FACSAria^TM^ III; BD Biosciences, CA, USA). The mice were administered CD83^+^CCR7^+^ NK cells (1 × 10^6^) at 4 days after immunization through an intravenous injection. Thereafter, at 4‐day intervals, mice were killed and the eyes, lymph nodes and spleens were harvested for H&E staining and flow cytometric analysis.

### 2.3 Anti‐IL‐18R antibody treatment

Anti‐IL‐18Rα antibody was purchased from R&D Systems Inc (Minneapolis, MN, USA). On day 8 after immunization, EAU mice were treated with anti‐IL‐18R antibody (10 µg/mouse, 100 µg/mouse, 450 µg/mouse)^41^, ^42^, ^43^ through an intravenous injection every day. At 8 days after administration of anti‐IL‐18R antibody, EAU mice were killed for harvesting of the eyes lymph nodes and spleens. H&E staining was used to assess the severity of retinal tissue damage. IgG (10 µg/mouse, 100 µg/mouse, 450 µg/mouse) was administered daily through an intravenous injection and was used as the negative control.

For evaluating anti‐IL‐18R antibody effects in in vitro experiments, NK cells were isolated from the eyes of EAU mice (10^6^/mL) and were pretreated with anti‐IL‐18R (1 µg/mL) for 24 hours. These NK cells were then washed twice with PBS and added to the DCs, T cells or combination of DCs and T cells.

### 2.4 Recombinant mouse interleukin‐18 binding protein treatment

On day 8 after immunization, EAU mice were treated with IL‐18 BP (Balb Co. Ltd., Beijing, China, 10 µg/mouse)^44^ through an intravenous injection every day. At 8 days after administration of IL‐18 BP, the eyes, lymph nodes and spleens of EAU mice were harvested. H&E staining was used to assess the severity of retinal tissue damage.

For evaluating IL‐18 BP effects in in vitro experiments, IL‐18 BP (1 µg/mL) was added to the co‐culture of NK cells and DCs or to the co‐cultures of NK cells, DCs and T cells.

### 2.5 NK cell isolation, culture and induction

CD3^‐^NK1.1^+^NK cells were isolated from splenic cells or ocular cells from EAU mice by a flow sorting instrument (BD FACSAria^TM^ III; BD Biosciences). These NK cells were cultured in RPMI 1640 (Sigma, St. Louis, MO, USA) containing 10% FBS (Atlanta Biologicals, Atlanta, GA, USA) and 250 IU/mL recombinant murine IL‐2 (R&D Systems Inc) at 37°C in a 5% CO_2_ incubator. To assess the effects of IL‐18 induction, isolated NK cells (10^6^/mL) were treated with IL‐18 (1 µg/mL) for 24‐48 hours.

### 2.6 Co‐culture of NK cells with DCs or DCs combined with T cells

Dendritic cells were isolated from spleens or ocular cells from EAU mice using a CD11c+isolation kit (Miltenyi Biotec, Germany). In brief, the spleen was minced and digested with collagenase D and DNase I at 37°C and filtered through a 70‐µmol/L nylon mesh. CD11c^+^DCs were then isolated using the manufacturer’s protocol and 33D1^+^CD11c^low^MHC‐II^low^ immature DCs or 33D1^+^CD11c^high^MHC‐II^high^CD11b^low^ mature DCs was further isolated by flow sorting instrument (BD FACSAria^TM^ III; BD Biosciences). Mature DCs used to active NK cells were treated with IRBP_1‐20_ (10 µg/mL) and PTX (10 µg/mL) overnight. After activation, cells were washed with phosphate buffer saline (PBS). NK cells isolated from the spleens of EAU mice on days 12‐16 after immunization with micro‐bead kits were co‐cultured overnight with isolated DCs (DC:NK=1:1). CD4^+^T cells were purified from the spleen of IRBP_1‐20_‐immunized B6 mice and were stimulated with IRBP_1‐20_ (10 µg/mL) in the presence of 1 × 10^7^ irradiated syngeneic spleen cells as APCs in the presence of IL‐2 (10 ng/mL). Antigen‐specific T cells were then obtained with use of magnetic beads and added to these co‐cultured cells for 24‐48 hours (T:DC+NK = 10:1). These cells were analysed using flow cytometry.

For anti‐IFN‐γR treatment, DCs were isolated from EAU mice and were pretreated with IRBP_1‐20_ (10 µg/mL) and PTX (10 µg/mL) overnight, followed by washing with PBS and addition of anti‐IFN‐γR (1 µg/mL) to the cells for 24 hours. These DCs were washed twice with PBS and were added to the NK, T or combination of NK and T cells. For anti‐IL‐18R treatment, NK cells were isolated from the eyes of EAU mice and were pretreated with anti‐IL‐18R (1 µg/mL) for 24 hours. These NK cells were then washed twice with PBS and were added to the DCs, T cells or combination of DCs and T cells. For anti‐IL‐27 neutralizing antibody treatment, anti‐IL‐27 neutralizing antibody (10 µg/mL) was added to the co‐culture system of DC and NK for 24 hours.

### 2.7 DCs depletion

To determine which cells produced IL‐18, CD45^+^ lymphocytes were isolated from the eyes of EAU mice. 33D1^+^CD11b^+^CD11c^+^MHC‐II^+^ DCs were labelled with corresponding fluorescent‐labelled antibodies and were depleted with a flow sorting instrument (BD FACSAria^TM^ III; BD Biosciences). Cells (1 × 10^6^) were cultured with 250 µL culture medium and stimulated with IRBP_1‐20_ (10 ng/mL) and PTX (10 ng/mL) as described previously.^3^, ^45^

### 2.8 Flow cytometric analysis

The cells from the eyes, lymph nodes and spleens were collected from naïve and EAU mice as previously described.^3^, ^46^ For analysis of immune cells within the eye, mice were selected with the same severity of inflammation in the eyes which were detected by Genesis‐D camera, and then, the eyes were pooled from 2 to 3 mice (4‐6 eyes) and removed the lens from these eyes. A single cell suspension was prepared by digestion for 10 minutes at 37°C with collagenase (1 mg/mL) and DNase (100 μg/mL) in RPMI‐1640. The ocular cells were then obtained following centrifugation. Aliquots of 1 × 10^6^ cells were stained with different monoclonal antibodies, according to protocols described for each of the antibodies. Cells were then analysed using FACSVerse and CellQuest data acquisition and analysis software (BD Biosciences). Fluorescent antibodies of CD3ε, NK1.1, CD69, NKG2D, NKG2A, CCR7, CD11c, MHC‐II, CD80, CD86, CD54, CD45, 33D1, CD11b, F4/80, ly6c, IFN‐γR, and IL‐18R conjugated with corresponding fluorescent dyes (eBioscience, San Diego, CA, USA) were used according to the manufacturer’s instructions. To assess intracellular cytokine expression including IFN‐γ, IL‐18, perforin and granzymes B, a cell stimulation cocktail (eBioscience) was used to stimulate the prepared cells for 5 hours at 37°C in a 5% CO_2_ environment. Corresponding antibodies were then applied.

### 2.9 ELISA analysis

Aqueous humour and serum were obtained from the mice. Aqueous humour and serum concentrations of IL‐18 and IFN‐γ were analysed with use of an ELISA kit of mouse IL‐18 or IFN‐γ (R&D Systems Inc). The assay was performed according to the instruction manual provided.

### 2.10 Statistical analysis

Data analyses were conducted using GraphPad Prism 5 (GraphPad Software, San Diego, CA, USA). Each experiment was performed in duplicate and replicated three times. Two‐tailed Student’s *t* tests or ANOVAs were applied to establish the presence of statistically significant differences between two groups or among the multiple sets of data respectively. For data failing to show homogeneity of variance, nonparametric Kruskal‐Wallis test was used for multiple independent samples. Data were presented as mean ± SEM and *P* < 0.05 (*), 0.01 (**) and 0.001 (***) were required for results to be considered statistically significant.

## 3 RESULTS

### 3.1 CD3^‐^NK1.1^+^CD83^+^CCR7^+^ NK cells are increased within the eyes in the EAU model

Induction of EAU was confirmed by the demonstration of its symptoms within the eyes, indicated by clinical and histopathological scores as previously described.^37^, ^38^ The disorganization of retinal tissue within the eyes of EAU mice and the dynamic clinical and histopathological scores of EAU mice are presented in Figure S1. Infiltrating CD3^‐^NK1.1^+^ NK cells within the inflamed eyes and spleens were found to increase on days 8‐12 (the initiation stage of EAU, Figure S1).

**Figure 1:**
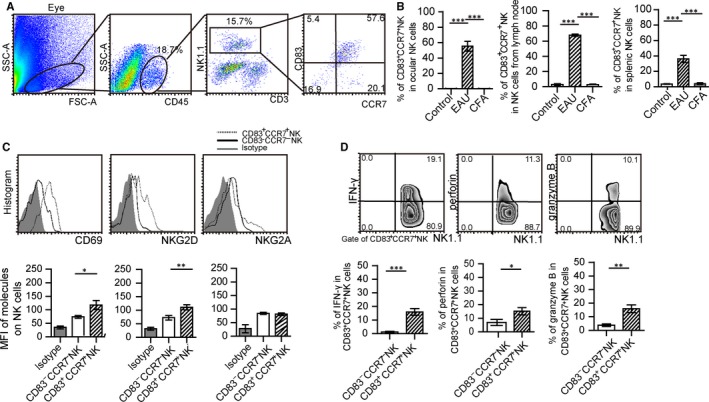
CD83^+^CCR7^+^ NK cells are increased within the eyes and spleens in the experimental autoimmune uveitis (EAU) model. (A) Representative pictures of CD83^+^CCR7^+^ NK cells in the eyes of EAU mice immunized on day 16. (B) Percent of CD83^+^CCR7^+^ NK cells in NK cells from the eyes, lymph nodes and spleens of EAU mice, control mice and complete freund's adjuvant (CFA) immunized mice (N = 15/group, values represent the mean ± SEM, ANOVA test, ***P < 0.001). (C) Expressions of CD69, NKG2D and NKG2A within CD3^‐^NK1.1^+^CD83^+^CCR7^+^ cells of EAU mice as compared with that of CD3^‐^NK1.1^+^CD83^‐^CCR7^‐^ (a representative result from three experiments, upper panel). Mean fluorescence value (MFI) of these molecules on CD3^‐^NK1.1^+^CD83^+^CCR7^+^ cells compared with these on CD3^‐^NK1.1^+^CD83^‐^CCR7^‐^ cells (N = 15/group and experiments were replicated three times, values represent the mean ± SEM, ANOVA test: *P < 0.05, **P < 0.01). (D) Expressions of IFN‐γ, perforin and granzyme B in CD3^‐^NK1.1^+^CD83^+^CCR7^+^ NK cells from EAU mice, as compared with that in CD3^‐^NK1.1^+^CD83^‐^CCR7^‐^ NK cells (N = 15/group and the experiment was replicated three times, values represent the mean ± SEM, two‐tailed Student’s *t* tests: *P < 0.05, **P < 0.01, ***P < 0.001)

In particular, approximately 57.6% ± 5.7% of infiltrated NK cells in the inflamed eyes were CD3^‐^NK1.1^+^CD83^+^CCR7^+^ cells, and 61.2% ± 5.7% of NK cells in the lymph nodes of EAU were CD3^‐^NK1.1^+^CD83^+^CCR7^+^ cells, while 38.6% ± 3.7% of NK cells within the spleen of EAU were CD3^‐^NK1.1^+^CD83^+^CCR7^+^ cells (Figure 1A,B). All of them were higher than those in the eyes, lymph nodes or spleen of control mice (Figure 1A,B). While activating signals, such as CD69 and NKG2D within CD3^‐^NK1.1^+^CD83^+^CCR7^+^ cells expressed higher than those signals in CD3^‐^NK1.1^+^CD83^‐^CCR7^‐^ cells, expression of the inhibitory receptor, NKG2A, was not significantly different between those two cells subsets (Figure 1C). These increased amounts of CD3^‐^NK1.1^+^CD83^+^CCR7^+^ cells were shown to express high levels of IFN‐γ, perforin and granzyme B as compared with that observed in CD3^‐^NK1.1^+^CD83^‐^CCR7^‐^ cells (Figure 1D). These results indicated that most of infiltrated NK cells within the eyes were CD83^+^CCR7^+^ NK cells which could express IFN‐γ.

### 3.2 Pathogenic role of CD83^+^CCR7^+^ NK cells within the EAU model

**Figure 2:**
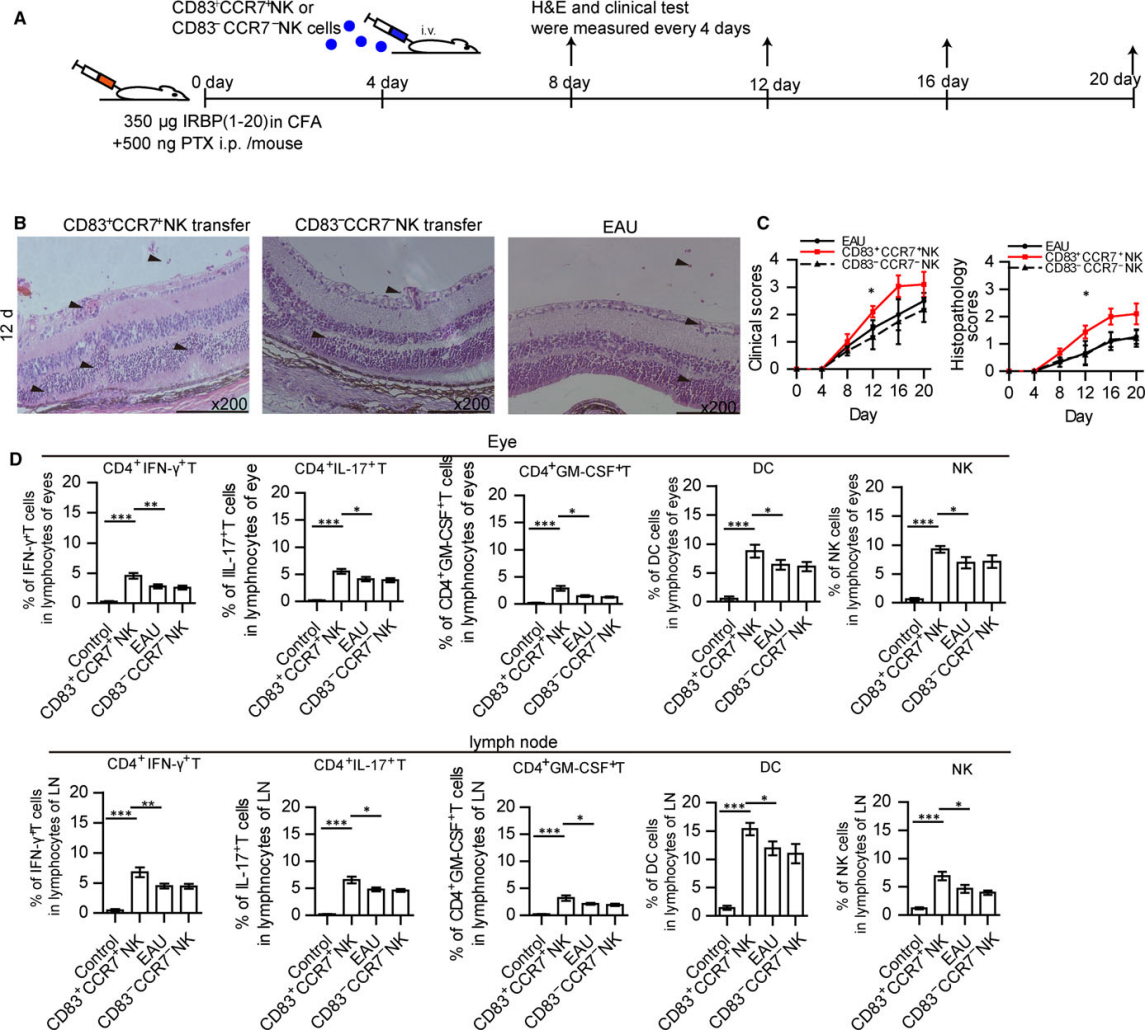
Function of CD83^+^CCR7^+^ NK cells in EAU mice. (A) Diagram of CD83^+^CCR7^+^ NK or CD83^‐^CCR7^‐^ NK cell transferred into immunized mice is as shown. (B) Histopathological damage of the eyes was assessed in CD83^+^CCR7^+^ NK cell transferred mice, compared with that of non‐treated EAU mice or CD83^‐^CCR7^‐^NK cell transferred mice by haematoxylin and eosin staining on the 12th day post‐immunization, and the respective histopathological and clinical scores were analysed every 4 days after immunization (C) (N = 15/group and the experiment was replicated three times, values represent the mean ± SEM, Kruskal‐Wallis test: *P < 0.05). (D) Percentage of CD4^+^ IFN‐γ^+^ T cells, CD4^+^ IL‐17^+^ T cells, CD4^+^ GM‐CSF^+^ T cells CD11b^+^CD11c^+^MHC‐II^+^DCs and CD3^‐^NK1.1^+^ NK cells in the eye and lymph nodes of mice receiving CD83^+^CCR7^+^NK or CD83^‐^CCR7^‐^NK cell transfers as compared with that of EAU mice. The gate of every picture is CD3^‐^NK1.1^+^CD83^+^CCR7^+^ (N = 5/group and the experiment was replicated three times, values represent the mean ± SEM, ANOVA test: *P < 0.05, **P < 0.01, ***P < 0.001)

To analyse the role of CD83^+^CCR7^+^NK cells in EAU, CD83^+^CCR7^+^NK or CD83^‐^CCR7^‐^NK cells were isolated from the inflamed spleen on days 12‐16 after immunization. These cells were then adoptively transferred into EAU mice that had been immunized 4 days prior (Figure 2A). The severity of symptoms of these mice was observed every 4 days after CD83^+^CCR7^+^ NK cells or CD83^‐^CCR7^‐^NK cell transfers. Retinal tissue damage and lymphocyte infiltration were present within the eyes of mice receiving this transfer of CD83^+^CCR7^+^NK cells on day 12 after immunization, and the retinal damage of these mice was more severe than that obtained in mice without cell transfer or with CD83^‐^CCR7^‐^ NK cell transfer (Figure 2B). Both clinical and histopathological scores of the eyes from mice receiving CD83^+^CCR7^+^ NK cell transfer were higher than those of mice without cell transfer or those receiving CD83^‐^CCR7^‐^ NK cell transfer (Figure 2C). The amount of lymphocyte subsets including CD4^+^IFN‐γ^+^ T cells, CD4^+^IL‐17^+^ T cells, CD4^+^GM‐SCF^+^ T cells, CD11c^+^ MHC‐II^+^ DCs and CD3^‐^NK1.1^+^ cells within the eyes of mice receiving CD83^+^CCR7^+^ NK cell transfer was greater than that in mice without a CD83^+^CCR7^+^ NK cell transfer or those with a CD83^‐^CCR7^‐^ NK cell transfer (Figure 2D). Similar phenomenon was found in the lymph nodes of the mice with CD83^+^CCR7^+^ NK cell transfer, but not in the spleen (Figure 2D and Figure S2). These data indicated that CD83^+^CCR7^+^ NK cells could affect the development of EAU and influence infiltrating T cells and DCs.

### 3.3 CD83^+^CCR7^+^ NK cells promote maturation of DCs

**Figure 3:**
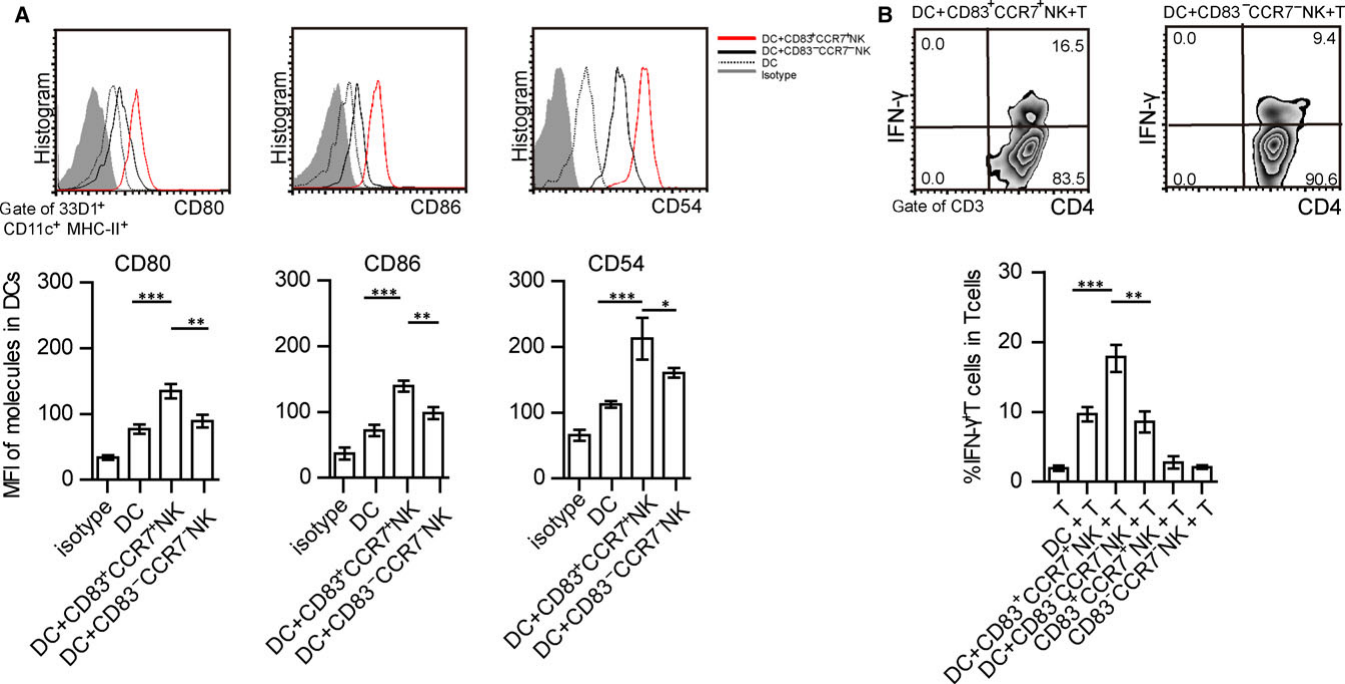
CD83^+^CCR7^+^ NK cells promote DC maturation and IFN‐γ secretion in T cells. (A) Expressions of CD80, CD86 and CD54 within immature 33D1^+^CD11c^+^MHC‐II^+^DCs co‐cultured with or without CD83^+^CCR7^+^ NK or CD83^‐^CCR7^‐^ NK cells. MFI of these molecules on dendritic cells (DCs), compared with DCs co‐cultured with CD3^‐^NK1.1^+^CD83^+^CCR7^+^ cells or DCs co‐cultured with CD3^‐^NK1.1^+^CD83^‐^CCR7^‐^ cells. (B) Expression of IFN‐γ in CD4^+^ T cells co‐cultured with DCs, DCs‐pretreated‐CD83^+^CCR7^+^ NK or DCs‐pretreated‐CD83^‐^CCR7^‐^ NK cells, or CD4^+^T cells co‐cultured with CD83^+^CCR7^+^ NK or CD83^‐^CCR7^‐^ NK cells (data presented in A and B represent that from three independent experiments, values represent the mean ± SEM, ANOVAs test: *P < 0.05, **P < 0.01, ***P < 0.001)

To further analyse whether CD83^+^CCR7^+^ NK cells could influence the status of DCs and T cells in vitro, CD83^+^CCR7^+^ NK cells from the eyes of EAU mice were isolated and co‐cultured with isolated 33D1^+^CD11c^low^MHC‐II^low^ DCs (immature DCs). DCs expressed high levels of CD80, CD86 and CD54 when co‐cultured with CD83^+^CCR7^+^ NK cells as compared with those lacking NK cells in the culture (Figure 3A) as well as those co‐cultured with CD83^‐^CCR7^‐^NK cells. Furthermore, to determine whether such mature DCs could affect the status of T cells, isolated CD4^+^ T cells were co‐cultured with DCs that were pretreated with either CD83^+^CCR7^+^ NK or CD83^‐^CCR7^‐^NK cells. These T cells, co‐cultured with DCs‐pretreated‐CD83^+^CCR7^+^ NK cells, expressed greater levels of IFN‐γ than those co‐cultured with non‐treated DCs or DCs‐pretreated‐CD83^‐^CCR7^‐^ NK cells (Figure 3B). However, neither CD83^+^CCR7^+^ NK cells nor CD83^‐^CCR7^‐^ NK cells alone were capable of affecting IFN‐γ secretion in T cells (Figure 3B). CD83^+^CCR7^+^ NK cells might play a pathogenic role to promote maturation of DCs which then enable T cells to express IFN‐γ.

Because CD83^+^CCR7^+^NK cells could secrete IFN‐γ to influence the statues of DCs.^21^ IFN‐γR on DCs was blocked by anti‐IFN‐γR antibody, and then these DCs were co‐cultured with CD83^+^CCR7^+^ NK cells. Expression levels of CD80, CD86 and CD54 in above DCs were lower than those without anti‐IFN‐γR antibody treatment, while no significant differences were observed in isolated DCs or anti‐IFN‐γR antibody pretreated isolated DCs (Figure S3A). Expression levels of IFN‐γ in T cells co‐cultured with CD83^+^CCR7^+^NK cells pretreated anti‐IFN‐γR antibody‐blocked‐DCs were also lower than that observed in CD83^+^CCR7^+^NK cells pretreated DCs (Figure S3B). In addition, IFN‐γR expression within DCs co‐cultured with CD83^+^CCR7^+^NK cells were higher than that of DCs co‐cultured with CD83^‐^CCR7^‐^NK cells (Figure S4).

### 3.4 Increasing levels of IL‐18 within the serum and aqueous humour of the EAU model may participate in CD83^+^CCR7^+^ NK cell induction

**Figure 4:**
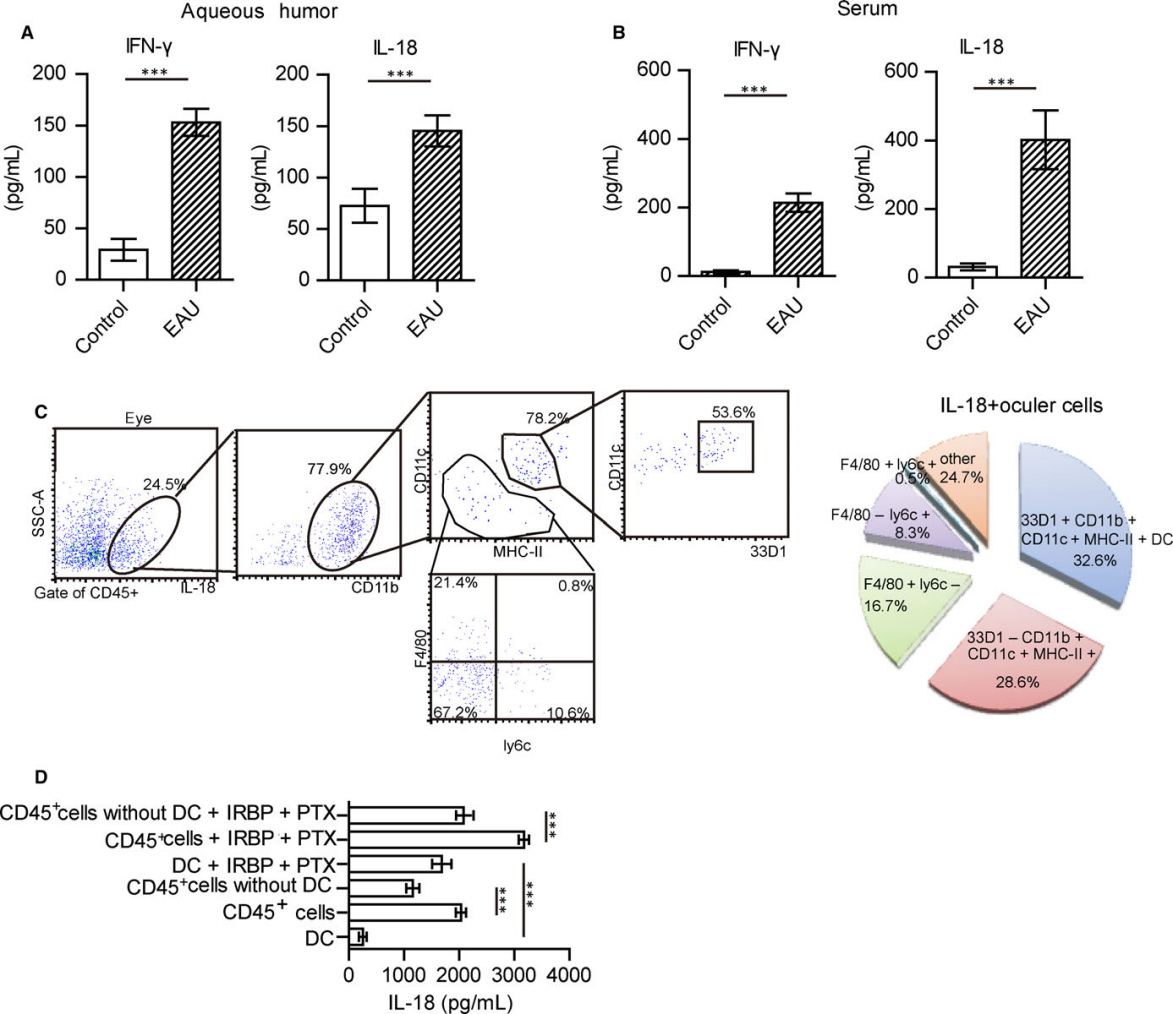
DCs represent the major subset responsible for secreting IL‐18. (A, B) Expressions of IFN‐γ and IL‐18 were present in the aqueous humour (A) and serum (B) of EAU mice (N = 20, values represent the mean ± SEM, two‐tailed Student’s *t* tests: ***P < 0.001). (C) Proportion of cell subsets in IL‐18 positive cells. IL‐18 positive cells were gated from ocular cells, and then 77.9% of IL‐18 + cells were CD11b positive cells, in which the percentage of 33D1^+^CD11b^+^CD11c^+^MHC‐II^+^, 33D1^‐^CD11b^+^CD11c^+^MHC‐II^+^, CD11b^+^F4/80^+^Ly6c^‐^, CD11b^+^F4/80^‐^Ly6c^+^, CD11b^+^F4/80^+^Ly6c^+^ were analysed. (D) With interphotoreceptor retinoid‐binding protein peptide (IRBP)_1‐20_ and pertussis toxin (PTX) stimulation or not, CD11c+DC, CD11c‐depleted magnetic isolated CD45+ cells from the eyes of EAU mice and CD45+ cells without deletion were cultured for 48 h. Data show the basal production of IL‐18 in the supernatants in non‐stimulated CD45+ lymphocytes or after stimulation with IRBP_1‐20_ (10 ng/mL) and PTX (10 ng/mL) (data from three independent experiments, values represent the mean ± SEM, ANOVA test: ***P < 0.001)

As IL‐18 has been reported to be an important factor involved in inducing subsets of CD83^+^CCR7^+^ NK cells,^21^ we examined whether IL‐18 could be detected in this EAU model. Concentrations of IL‐18, as well as IFN‐γ, were significantly increased both in the aqueous humour of the inflamed eyes (Figure 4A) and serum (Figure 4B) of EAU mice as compared with their respective controls. Results obtained from in vitro experiments substantiated that IL‐18 promoted the expression of CD83 and CCR7, as well as IFN‐γ production within NK cells (Figure S5A,B). With IL‐18 induction, the percent of CD83^+^CCR7^+^NK cells in NK also increased (Figure S5C).

When IL‐18 binding protein (IL‐18 BP) was injected into mice to neutralize IL‐18, the symptoms of EAU and percent of CD83^+^CCR7^+^NK cells within the eyes were decreased (Figure S6A‐C). Furthermore, the expression of IL‐18R within CD83^+^CCR7^+^NK or CD83^‐^CCR7^‐^NK cells was also detected to show that levels of IL‐18R expression within infiltrated CD83^+^CCR7^+^NK cells were higher as compared with that of CD83^‐^CCR7^‐^NK cells (Figure S7).

### 3.5 DCs participated in the production of IL‐18 in EAU

As IL‐18 is reported to be produced primarily by macrophages, neutrophils and DCs,^19^, ^22^, ^24^ we next examined the status of macrophages, neutrophils and DCs in EAU. The percent of CD11b^+^CD11c^+^MHC‐II^+^ DCs, CD11b^+^ly6c^‐^F4/80^+^ macrophages, CD11b^+^ly6c^+^F4/80^+^ neutrophil/granulocytes and CD11b^+^ly6c^+^F4/80^‐^ monocytes/neutrophils were increased in the inflamed eyes, lymph nodes and spleens of EAU mice (Figure S8A). DCs were reported to exist in the peripheral margins and juxtapapillary areas of the retina, and specific express 33D1+.^47^ 33D1^+^CD11b^+^CD11c^+^MHC‐II^+^ DCs from the inflamed eyes accounted for a large proportion of IL‐18 secreting cells (Figure 4C). DCs from inflamed spleens, or lymph nodes also accounted for the most proportion of IL‐18 secreting cells (Figure S8B). IL‐18 positive DCs from the eyes were also detected (Figure S8C). The status of IL‐18^+^ DCs was analysed with flow cytometry. These DCs expressed higher levels of CD80, CD86 and CD54 as compared with that of IL‐18^‐^ DCs (Figure S8D). Such results indicated that these IL‐18 secreting DCs had matured.

To further identify the main source of IL‐18 in the eyes, we isolated CD45^+^ cells and further depleted 33D1^+^ DCs. The level of IL‐18 in the supernatant of cell cultures was assessed by ELISA. Depletion of 33D1^+^ DCs exerted the strongest negative effect on the basal release of IL‐18 (2201.4 ± 58.29 pg/mL in total CD45^+^ cells vs 1283.48 ± 64.3 pg/mL in CD11c^+^ DCs depleted CD45^+^ cells) (Figure 4D). With antigen stimulation, the level of IL‐18 in purified 33D1^+^ DCs was higher than that without stimulation (Figure 4D). With antigen stimulation, IL‐18 from depleted CD45+ cells was also increased as compared with that observed in those cultures without depletion (Figure 4D). These results indicated that DCs represented the main source of IL‐18 in the eyes.

**Figure 5:**
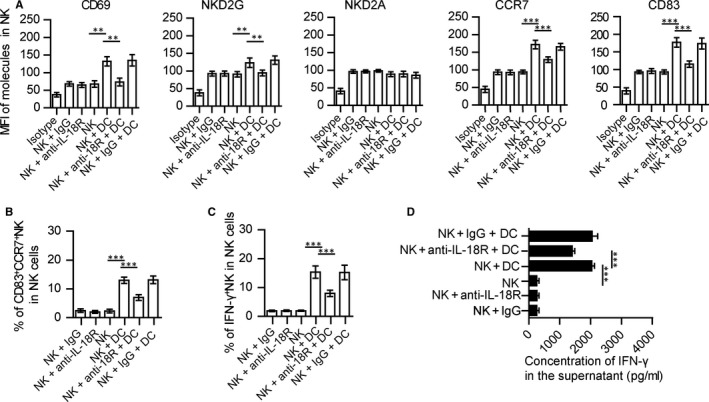
DCs activate CD83^+^CCR7^+^ NK cells in vitro. CD3^‐^NK1.1^+^ NK cells and 33D1^+^ CD11b^+^CD11c^+^MHC‐II^+^DCs were isolated from the inflamed eyes of EAU mice on days 12‐16 after immunization. DCs were then induced to maturity by IRBP and PTX and co‐cultured with NK cells for 24‐48 h (DC:NK = 1:1). (A) Expressions of CD69, CD83, NKG2D, NKG2A and CCR7 in NK cells, NK cells co‐cultured with mature DCs, anti‐IL‐18R‐pretreated‐NK cells co‐cultured with mature DCs or IgG‐pretreated‐NK cells co‐cultured with mature DCs. (B) Percent of CD83^+^CCR7^+^ NK cells in NK cells were determined after NK cells co‐cultured with or without matured DCs or anti‐IL‐18R‐pretreated‐NK cells co‐cultured with matured DCs, IgG‐ pretreated‐NK cells co‐cultured with matured DCs for 24 h. (C) Expression of IFN‐γ in NK cells with or without DCs co‐culture or anti‐IL‐18R‐pretreated‐NK cells co‐cultured with DCs, IgG‐pretreated‐NK cells co‐cultured with DCs were determined. (D) Concentration of IFN‐γ in the supernatant of various sets of cell culture as indicated (all data are from three independent experiments, N = 15, values represent the mean ± SEM, ANOVA test: *P < 0.05, **P < 0.01, ***P < 0.001)

To assess whether these matured DCs from the eyes could affect the activation of NK cells. NK cells and DCs were isolated and co‐cultured for 24‐48 hours. Expressions of CD69, NKG2D, CCR7 and CD83 within NK cells co‐cultured with DCs were increased as compared with those co‐cultured without DCs (Figure 5A). The percentage of CD83^+^CCR7^+^ NK cells increased after being co‐cultured with DCs (Figure 5B). Moreover, the expression (Figure 5C) and concentration (Figure 5D) of IFN‐γ in NK cells were also increased in co‐culture’s medium of NK and DC cells, compared to NK cells cultured alone.

With IL‐18 BP treatment in vitro, NK cells in co‐cultures of DCs and NK cells could not be activated, compared with those without IL‐18 BP treatment (Figure S6D,E). The level of IFN‐γ in NK cells was lower than those without treatment. Thus, IL‐18 secreted from DCs might promote NK to secret IFN‐γ. IL‐27 also was reported to be secreted from DC to promote NK to express IFN‐γ.^9^ With IL‐27 neutralizing antibody treatment, or IL‐27 neutralizing antibody and IL‐18BP combined treatment, the percentage of IFN‐γ^+^ NKs and the concentration of IFN‐γ in the DC‐NK co‐cultured medium decreased (Figure S6E,F). With IL‐27 neutralizing antibody and IL‐18BP combined blockage, the production of IFN‐γ in NK cells is significantly lower than either of those blockage (Figure S6E,F).

### 3.6 Anti‐IL‐18R antibody decreases the percent of CD83^+^CCR7^+^ NK cells in vitro

To further evaluate whether the effect of DC in NK cells is through IL‐18, we blocked the IL‐18R in NK cells with anti‐IL‐18R antibody, and then co‐cultured with matured DCs isolated from EAU mice. The expressions of CD69, CD83, CCR7 and NKG2D within anti‐IL‐18R‐treated‐NK cells co‐cultured with DCs were decreased as compared with non‐treated or IgG‐treated‐NK cells (Figure 5A). With anti‐IL‐18R antibody treatment, the percent of CD83^+^CCR7^+^ NK cells (Figure 5B) and expression (Figure 5C) and secretion (Figure 5D) of IFN‐γ in NK cells were all decreased as compared with those non‐treated or IgG‐treated‐NK cell. Blocking of IL‐18R also reduced the effects of DC on CD83^+^CCR7^+^NK cells (Figure S9A,B), but had no effect on CD83^‐^CCR7^‐^NK cells (Figure S9C,D).

### 3.7 Anti‐IL‐18R antibody treatment relieves EAU symptoms and decreases NK cell infiltration within the inflamed eyes

**Figure 6:**
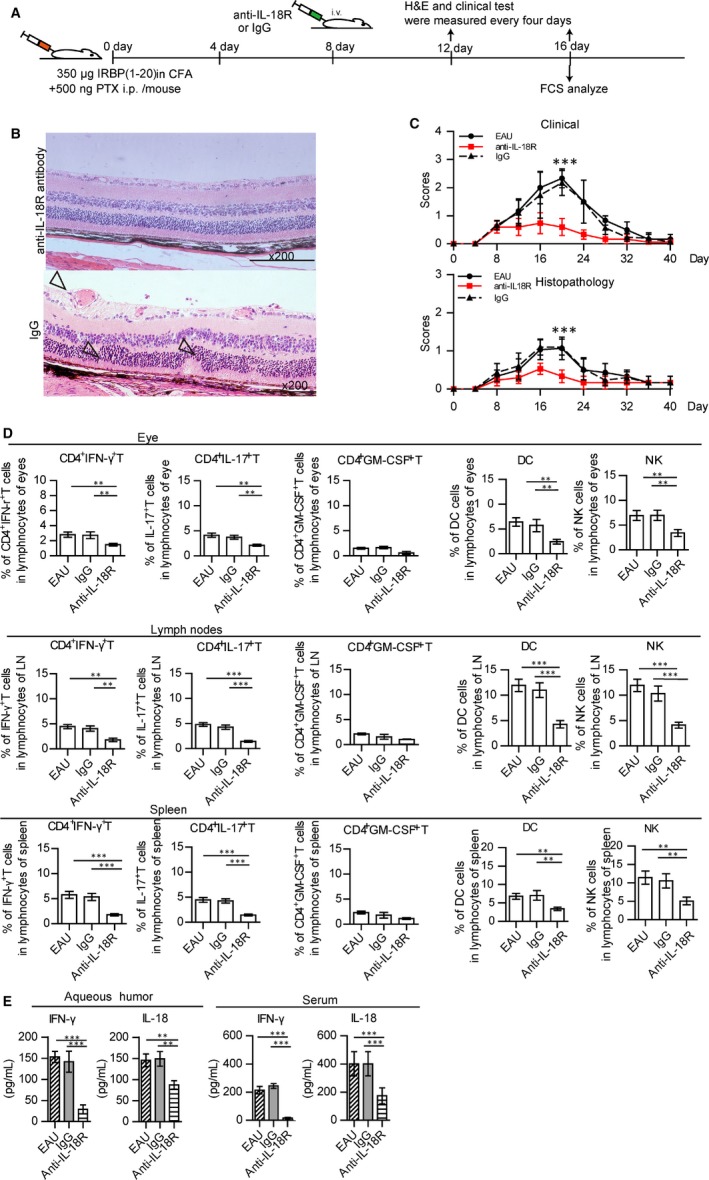
Anti‐IL‐18R antibody relieved symptoms of EAU and decreased inflammatory factors in EAU. (A) Diagram of anti‐IL‐18R antibody treatment in EAU. Anti‐IL‐18R antibody treatment was administered daily starting at 8 days after immunization, H&E staining and clinical test was performed every 4 days, and flow cytometry analysis on day 16. (B) Example of histopathology of a representative eye section from anti‐IL‐18R‐ or IgG‐treated mouse (haematoxylin and eosin, original magnification, ×200). Vasculitis and photoreceptor folding within the retina were present within the inflamed eye (hollow arrows). (C) Histopathological and/or clinical scores of EAU, anti‐IL‐18R‐treated and IgG‐treated mice (three experiments with N = 3/group values represent the mean ± SEM Kruskal‐Wallis test, ***P < 0.001). (D) Percent of CD4^+^ IFN‐γ^+^ T cells, CD4^+^ IL‐17^+^ T cells, CD4^+^ GM‐CSF^+^ T cells, NK cells and DCs as determined within the eyes, lymph nodes (LN) or spleens of EAU, anti‐IL‐18R antibody‐treated and IgG‐treated mice. (E) Expressions of IFN‐γ and IL‐18 in the aqueous humour and serum of EAU, anti‐IL‐18R antibody‐treated and IgG‐treated mice (data of D, E were from three experiments with N = 3/group, values represent the mean ± SEM, ANOVA test: *P < 0.05, **P < 0.01, ***P < 0.001)

To assess whether anti‐IL‐18R antibody could affect the symptoms of EAU, anti‐IL‐18R antibody was administered through the tail vein of EAU mice that had been immunized for 8 days. At 8 days after anti‐IL‐18R antibody injection, ocular cells and splenic cells were harvested for analysis (Figure 6A). While there was no obvious retinal tissue damage in response to the anti‐IL‐18R antibody treatment (Figure 6B), the outer nuclear layer of retinal tissue in these mice with IgG treatment was deformed (Figure 6B). Histopathological and clinical scores of the eyes in anti‐IL‐18R‐treated‐mice were decreased as compared with that of EAU and IgG‐treated mice (Figure 6C). Although the symptoms of EAU were alleviated with higher doses of anti‐IL‐18R antibody, no significant difference of the symptomatic remission was shown between higher and lower dose of anti‐IL‐18R treatment (Figure S10).

Anti‐IL‐18R antibody treatment also significantly decreased the percent of CD4^+^ IFN‐γ^+^ T cells, CD4^+^ IL‐17^+^ T cells, CD3^‐^ NK1.1^+^ NK and CD11b^+^ CD11c^+^ DCs cells within the eyes, lymph nodes and spleens as compared with non‐treated EAU mice (Figure 6D). Concentrations of IFN‐γ and IL‐18 within the aqueous humour and serum were decreased in anti‐IL‐18R‐treated‐mice as compared with that observed in untreated mice (Figure 6E).

We also found that anti‐IL‐18R‐pretreated isolated NK cells failed to increase the expressions of CD80, CD86 and CD54 on DCs, compared with those without anti‐IL‐18R treatment (Figure S11A). In addition, DCs pretreated with anti‐IL‐18R treated NK cells failed to affect IFN‐γ expression in T cells (Figure S11B). These data indicated that anti‐IL‐18R antibody could suppress the activation of NK cells and DCs along with IFN‐γ expression in T cells.

## 4 DISCUSSION

Uveitis is a serious inflammatory disease that can result in visual disability and blindness.^1^ The primary basis for recurrent uveitis involves disorders of the immune system^1^, ^2^ with uveitogenic antigen‐specific T cells serving as crucial effectors driving this disorder of the immune system to result in tissue damage.^2^, ^48^, ^49^, ^50^ The severity of EAU is augmented by NK cells which were reported to play a pathological role in uveitis.^3^, ^4^, ^6^, ^7^, ^8^, ^9^ Regulating the status of NK cells could modulate the process of EAU. Our current data provide further evidence that the increased CD3^‐^NK.1.1^+^CD83^+^CCR7^+^ cells in EAU play a pathological role in the development of EAU. The increased levels of CCR7 within such NK cells appear to be related with NK cell migration,^51^ while enhanced levels of CD83, NKG2D and CD69 expressions within these NK cells are associated with NK cells activation. In this study, these increased CD83^+^CCR7^+^ NK cells aggravate EAU symptoms by increasing the activation of DCs and T cells. In contrast, CD83^‐^CCR7^‐^NK cells do not.

IL‐18 is a cytokine that belongs to the IL‐1 super‐family and is an inflammatory factor in many diseases.^31^, ^36^, ^52^, ^53^, ^54^ Increases in intraocular levels of IL‐1β, IL‐2, TNF‐α, IFN‐γ, IL‐6, IL‐8 and MCP‐1 have all been found to be associated with uveitis.^55^, ^56^ However, the mechanisms of IL‐18 as related to uveitis remain unknown. In our study, we now provide evidence indicating that IL‐18 is a pathogenic factor in EAU and provide a description for some possible mechanisms of IL‐18 in uveitis. IL‐18 can induce NK cell activation to secrete IFN‐γ and increase expression levels of CCR7, CD83, NKG2D and CD69 on NK cells. Thus, IL‐18 has the capacity to promote NK cell activity and migration to inflammatory sites, where it may then function as a critical factor in EAU through induction of CD83^+^CCR7^+^ NK cells.

IL‐18 is mainly produced by macrophages, neutrophils and DCs.^19^, ^22^, ^24^ In our experiments, we found that macrophages, neutrophils and DCs were all increased in EAU and secreted IL‐18. But macrophages and neutrophils were not the primary increasing cells in the inflamed eyes of EAU. DCs as an important pathogenic factor for EAU, might participate in producing IL‐18 to promote pathogenic CD83^+^CCR7^+^NK cell activation in the eyes of EAU. And then, these NK cells migrate into lymph nodes to promote DC maturation and T‐cell activation.^9^, ^14^, ^51^ Additionally, our findings demonstrate that DCs in EAU promote pathogenic NK cell activation, indicating a novel pathologic mechanism of DCs in EAU. DCs are activated and migrate to the lymph nodes to activate T cells, or activate the small resident population of NK cells to produce IFN‐γ, which in turn induces the DCs to produce IL‐18. In the inflamed eyes, activated DCs might also secret IL‐18 to induce CD83^+^CCR7^+^NK cells, which secrete IFN‐γ to influence the statues of DCs in turn.

IL‐18 is also known as an interferon‐gamma inducing factor. In this capacity, it functions by binding to the interleukin‐18 receptor which then activates NK cells.^21^, ^26^, ^53^, ^57^ Our findings show that IL‐18 secreted by DCs stimulated NK cell activation, which in turn promoted DCs maturation. These mature DCs then induced T cells to secrete IFN‐γ. When IL‐18/IL‐18R interactions were blocked, these DCs could not activate NK cells in vitro. Thus, IL‐18 might play a key role in inducing the cycle of DC maturation and NK activation. An axis may exist between DC‐NK interactions to regulate Th1 responses in this EAU model. In support of this eventuality are the findings demonstrating that an initial DC‐NK interaction controls an adaptive Th17 response and limits tissue‐specific autoimmunity through an innate IFN‐γ‐IL‐27 axis.^9^ Our data provide support for this possibility, as indicated by the presence of a regulatory role of T‐cell activation through DC‐NK interactions in EAU. With anti‐IL‐27 neutralizing antibody or IL‐18 BP treatment, the concentration of IFN‐γ in the medium co‐culture of DC and NK decrease. There is no significant difference on the production of IFN‐γ in NK cells between anti‐IL‐27 and IL‐18 BP treatment. With IL‐27 neutralizing antibody and IL‐18BP combined blockage, the production of IFN‐γ in NK cells is significantly lower than either of those blockage. There may be a synergy between IL‐27 and IL‐18. The relationship between IFN‐γ and IL‐27 or IL‐18 needs further study. Additionally, another axis or factors regulating DC‐NK interaction in EAU may be present, which will be investigated in further studies within our laboratory. In addition, IFN‐γ has been recognized to have pleiotropic effects in autoimmunity.^58^, ^59^, ^60^ The function of IFN‐γ in autoimmune disease may be related to its dosage and time.^61^, ^62^ The mechanism of IFN‐γ in EAU needs further studying.

The treatment of uveitis remains a challenging undertaking and an area of active research.^1^, ^63^, ^64^ Conventional therapy involves use of corticosteroids as a first‐line therapy for patients with active uveitis, however, long‐term corticosteroid treatment can result in serious systemic and ocular side effects.^2^, ^64^ Alternatively, immune‐modulatory therapy drugs administered as steroid‐sparing agents have shown effective clinical results for both systemic diseases and ocular inflammatory diseases and it is anticipated that the development of novel immunomodulatory drug therapies will replace conventional drugs to achieve enhanced therapeutic effects.^63^, ^64^, ^65^ While immunotherapy represents the main treatment protocol for autoimmune uveitis, it is obvious that further studies are needed to identify the most effective targets.^63^ In this study, we show that anti‐IL‐18R antibody treatment decreases the severity of retinal damage and the number of infiltrated T cells, NK cells and DCs in the eyes, lymph nodes and inflamed spleens in EAU mouse model. Moreover, this treatment reduces the levels of IFN‐γ and IL‐18 in the serum and aqueous humour in these mice. The mechanisms involved in these effects appear to entail a blocking of IL‐18/IL‐18R interactions. Such an effect prevents the IL‐18‐induced NK cell activation to reduce CD4^+^ T cells in EAU. As IL‐18R is also expressed in T cells, DCs and other cells, blocking IL‐18R with use of an anti‐IL‐18R antibody could directly influence the statues of T cells. Anti‐IL‐18R antibody might serve as a possible therapeutic candidate for the treatment of autoimmune uveitis. However, anti‐IL‐18R treatment at the initial stage of uveitis in our study might have some limits. As at the initial stage of uveitis, the symptom of uveitis cannot be detected by clinical methods, the effect of anti‐IL‐18R on uveitis patients is difficult to be evaluated. The dose and the time of anti‐IL‐18R administration need further studying.

## CONFLICT OF INTEREST

All the authors have declared there are no financial conflicts of interest with regard to this work.

## AUTHORS’ CONTRIBUTION

QF and WL designed the study, performed experiments, analysed the data, drafted and revised the manuscript; XM performed the animal experiments and flow cytometry; XW, NS performed the cell cultures, organized the samples and performed the experiments; YL and JX performed statistical analysis of the data; YS generated the figures and tables. All the authors approved the final version of this manuscript.
