# Supplementary figures and images for: CD83+CCR7+ NK cells induced by interleukin 18 by dendritic cells promote experimental autoimmune uveitis
Source: J Cell Mol Med. 2018 Dec 8;23(3):1827–39. doi: 10.1111/jcmm.14081 (PMC6378215; doi:10.1111/jcmm.14081)

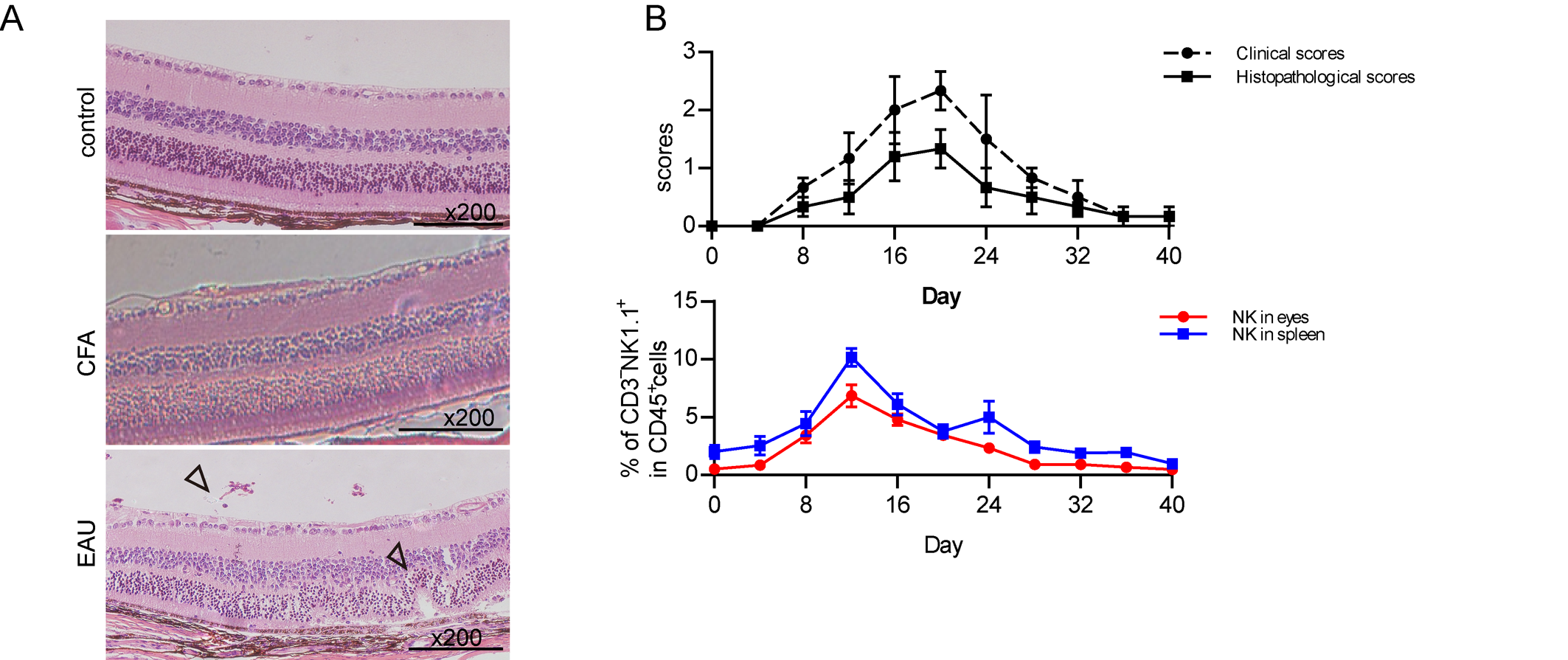

Supplement: Supplementary file 1 [file JCMM-23-1827-s001.tif]

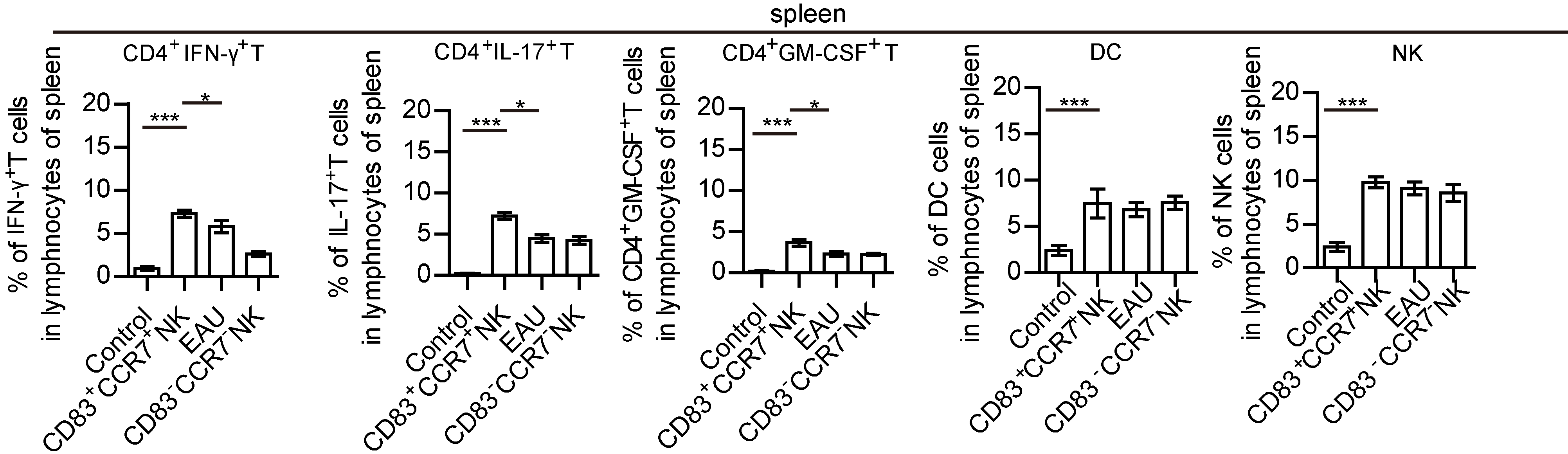

Supplement: Supplementary file 2 [file JCMM-23-1827-s002.tif]

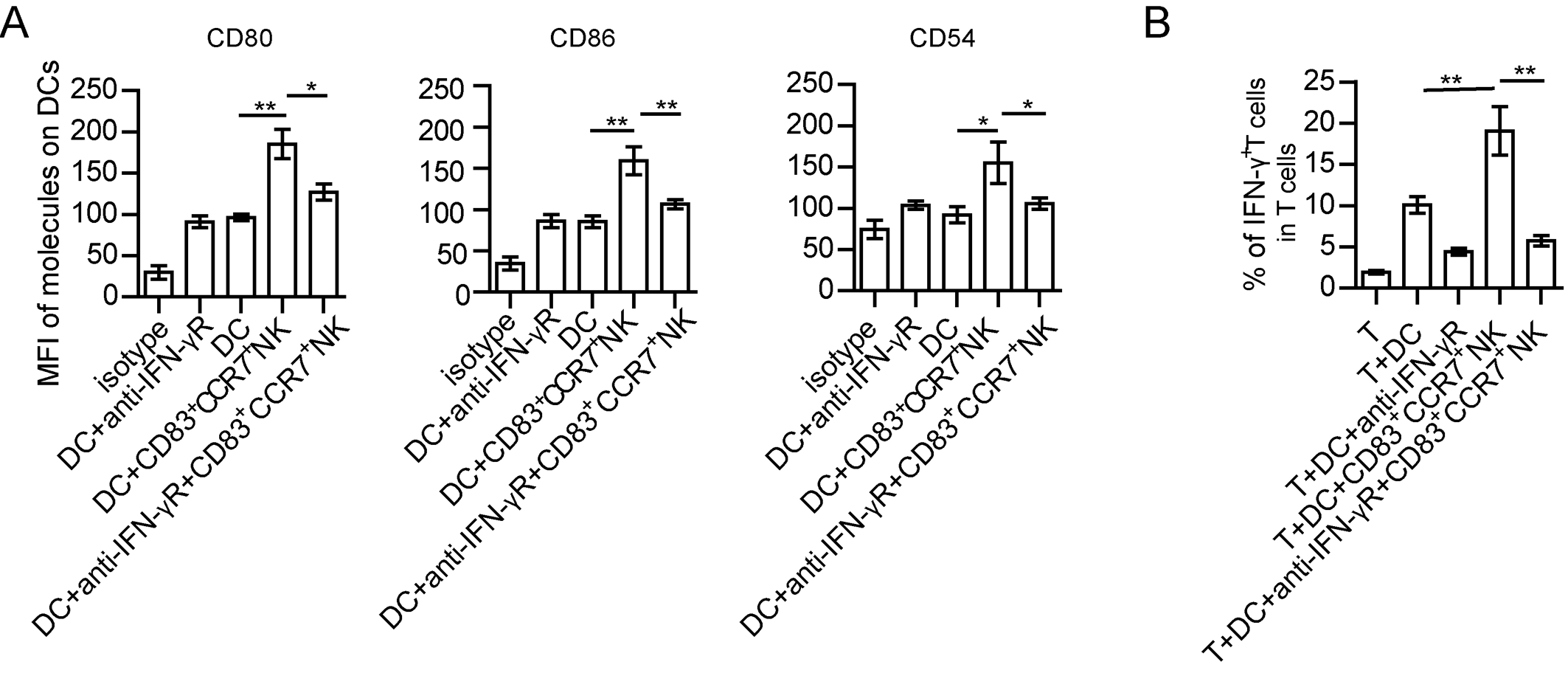

Supplement: Supplementary file 3 [file JCMM-23-1827-s003.tif]

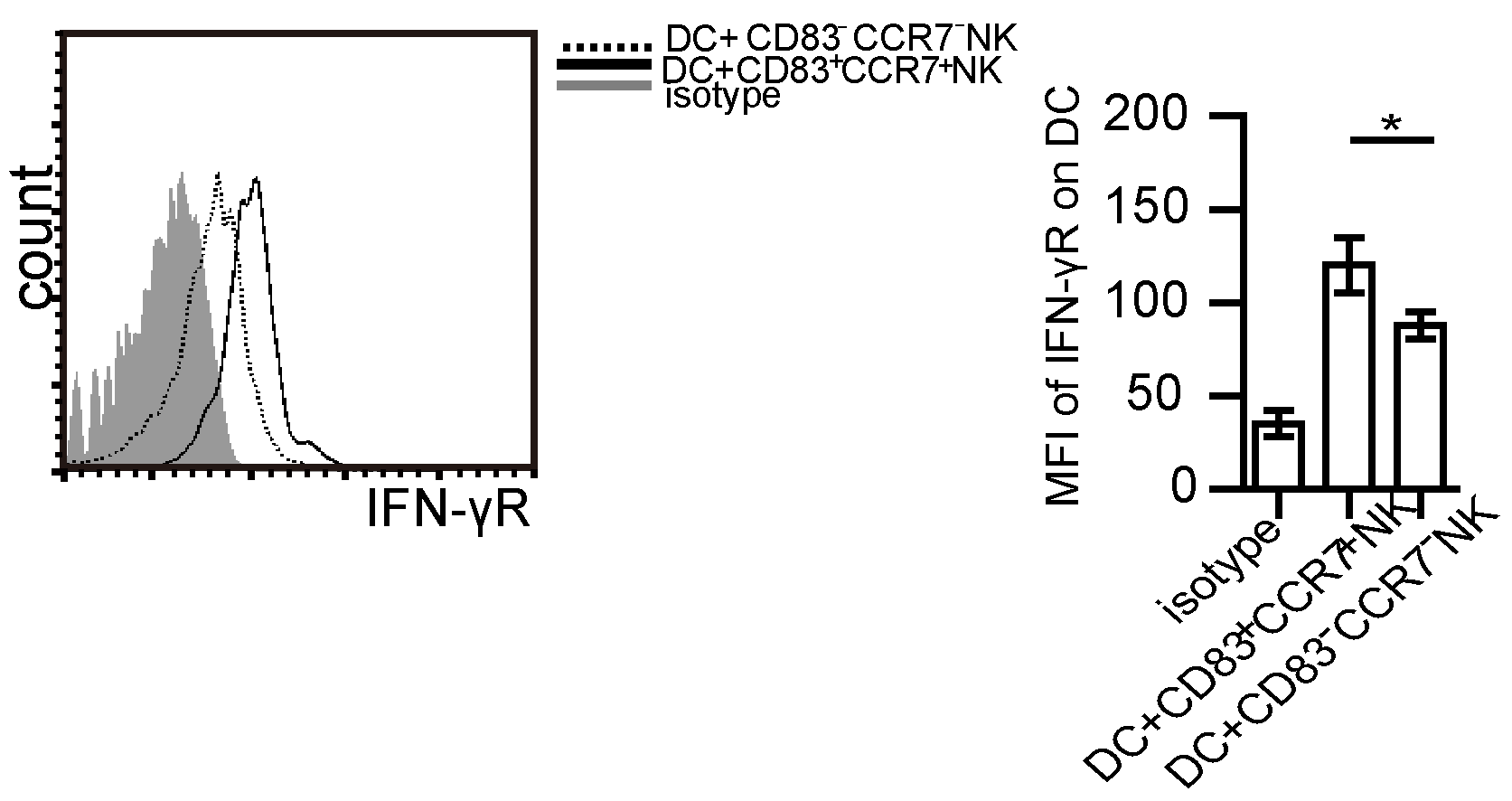

Supplement: Supplementary file 4 [file JCMM-23-1827-s004.tif]

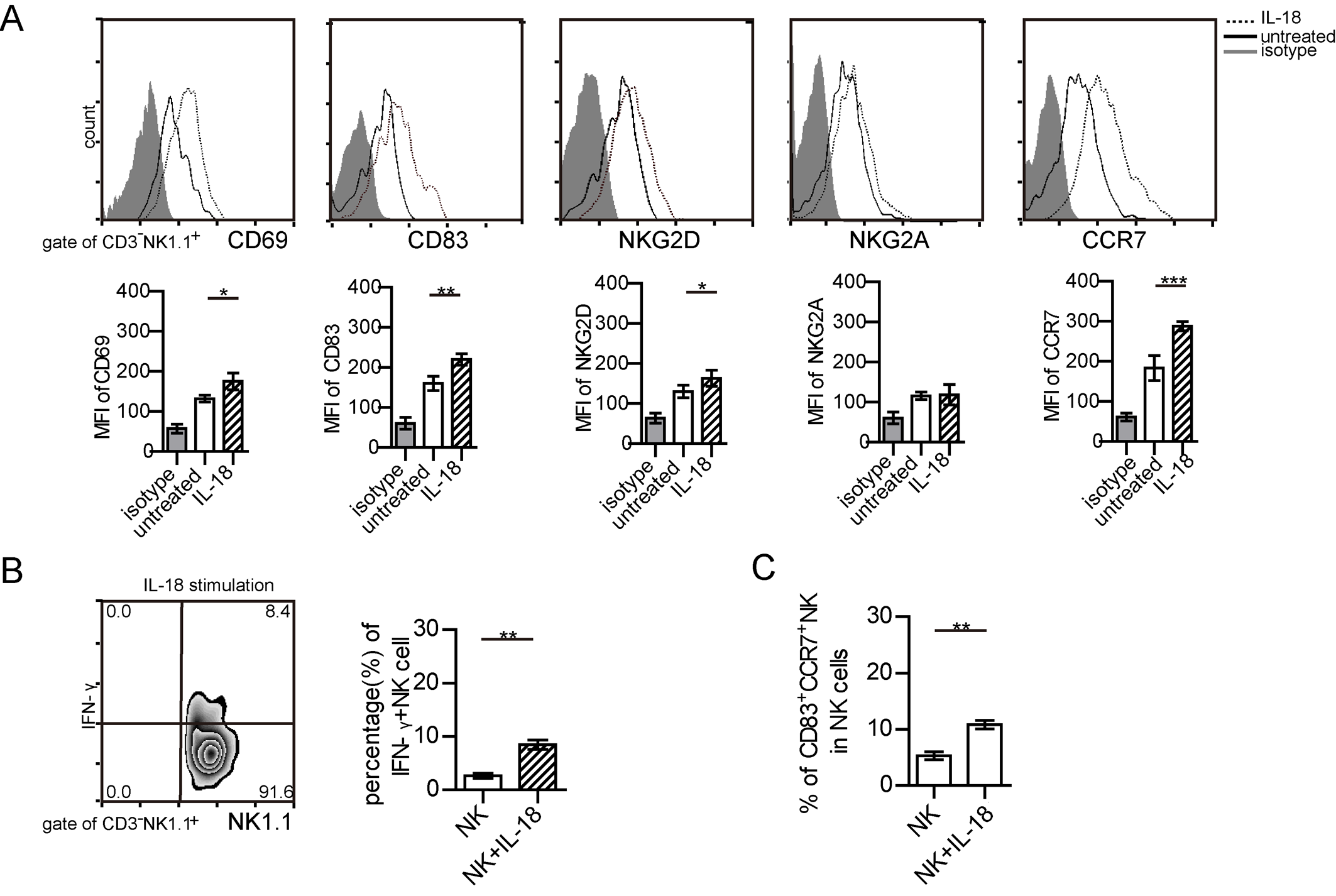

Supplement: Supplementary file 5 [file JCMM-23-1827-s005.tif]

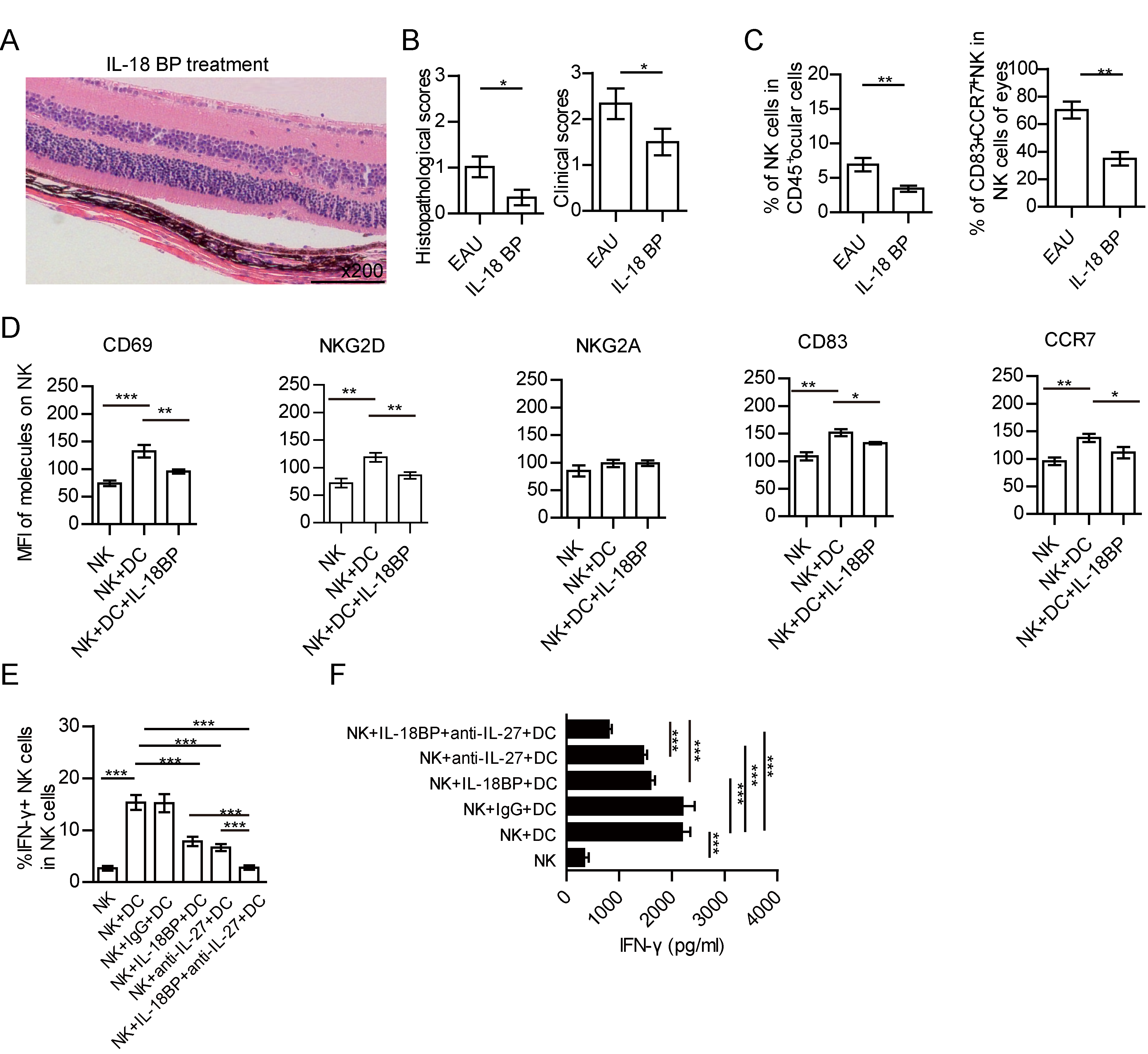

Supplement: Supplementary file 6 [file JCMM-23-1827-s006.tif]

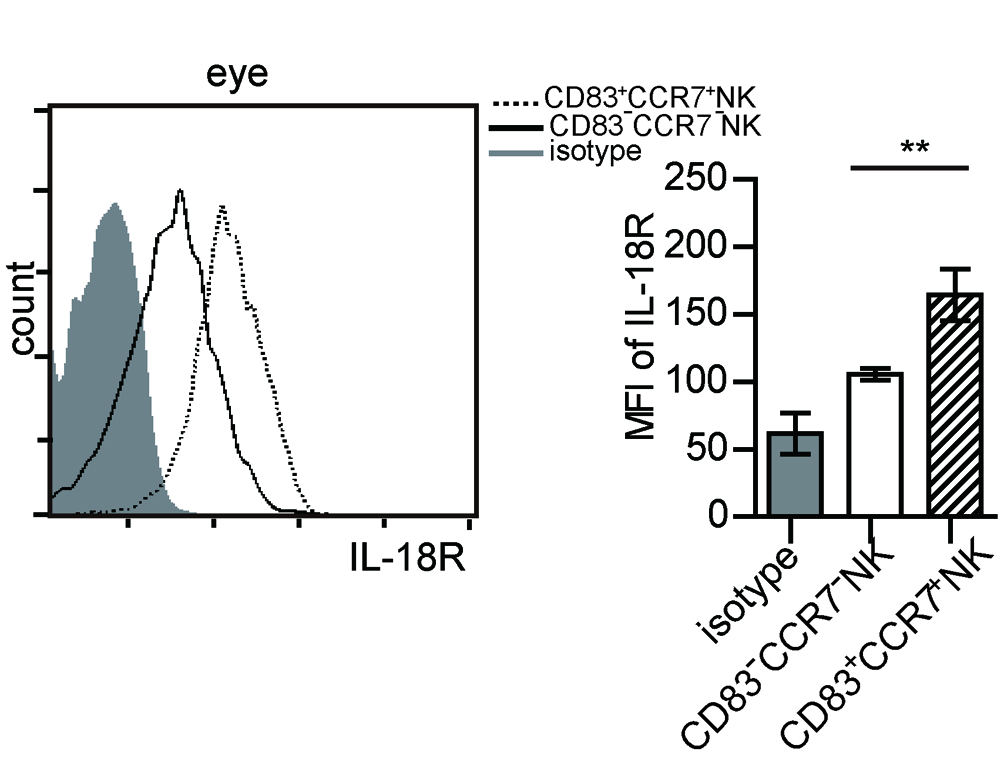

Supplement: Supplementary file 7 [file JCMM-23-1827-s007.tif]

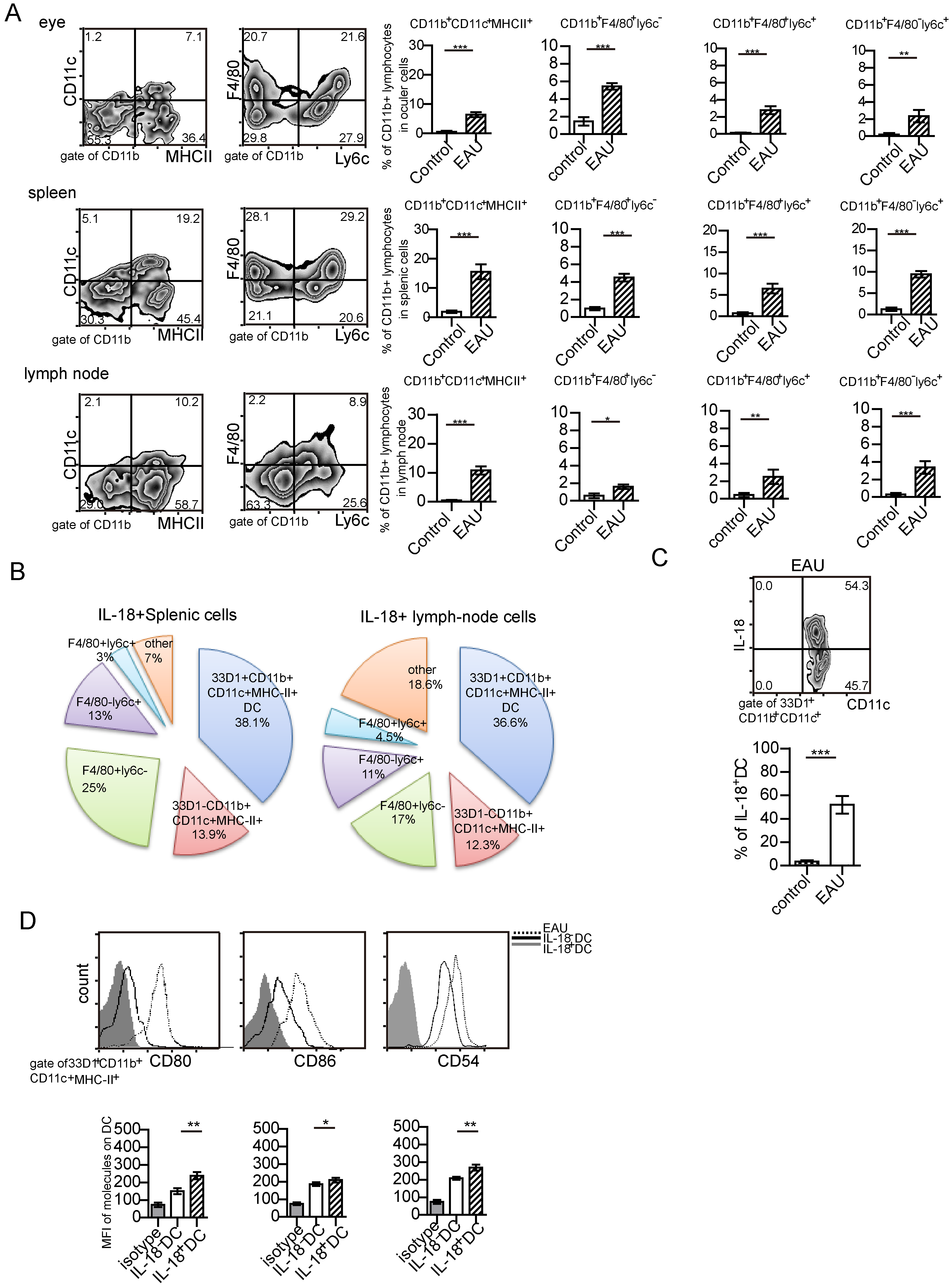

Supplement: Supplementary file 8 [file JCMM-23-1827-s008.tif]

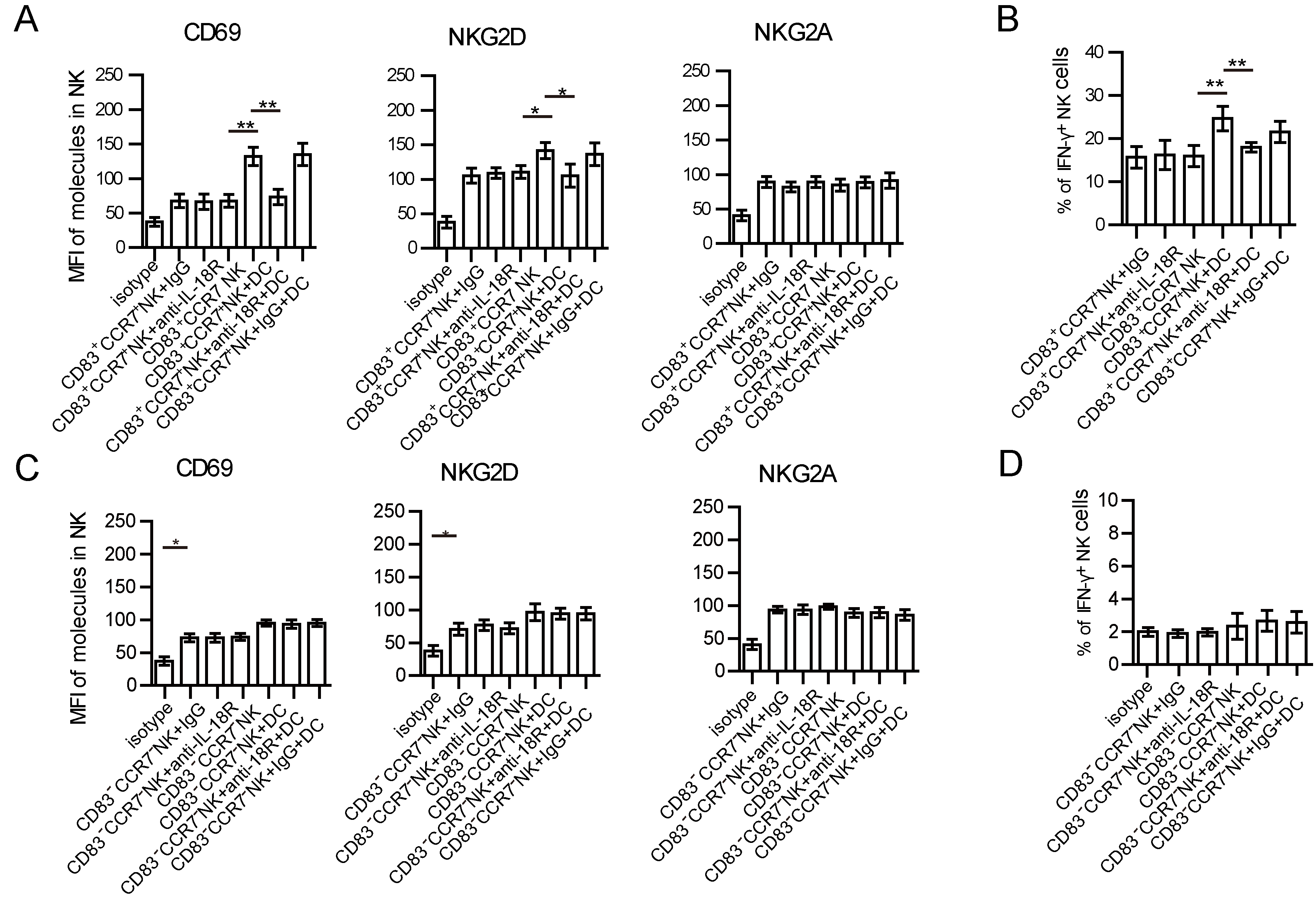

Supplement: Supplementary file 9 [file JCMM-23-1827-s009.tif]

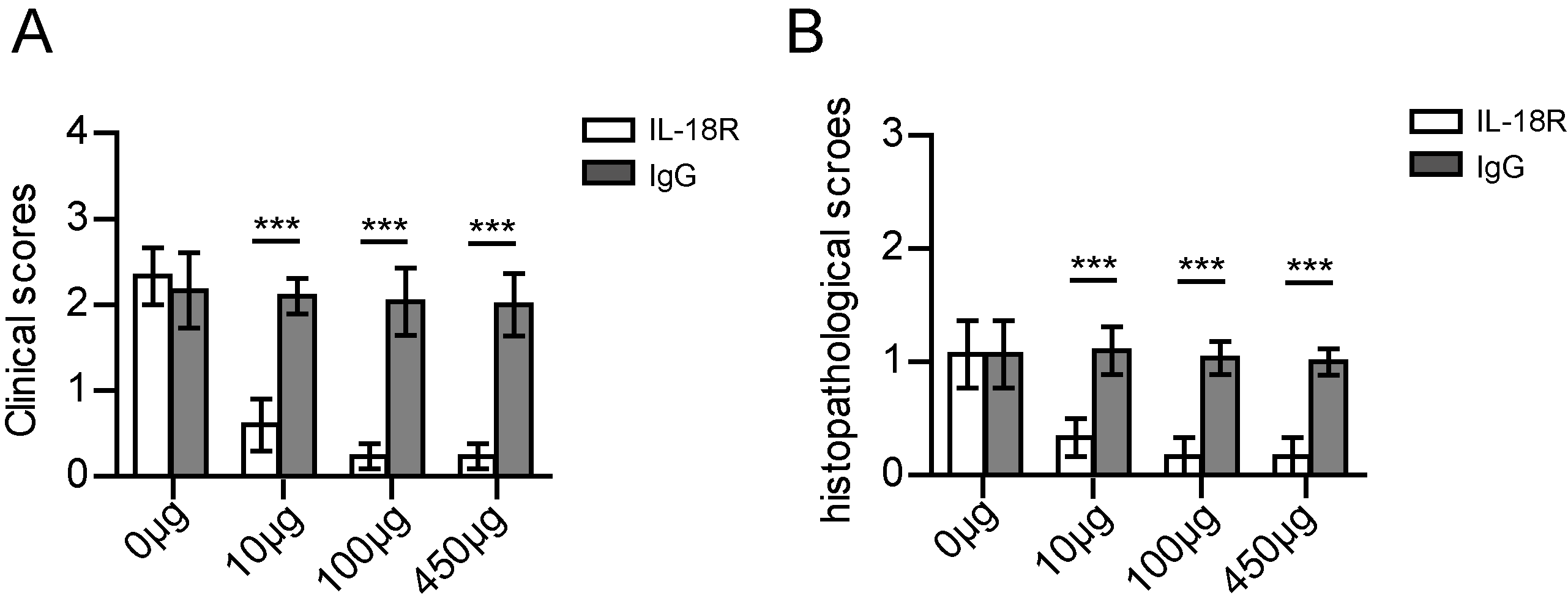

Supplement: Supplementary file 10 [file JCMM-23-1827-s010.tif]

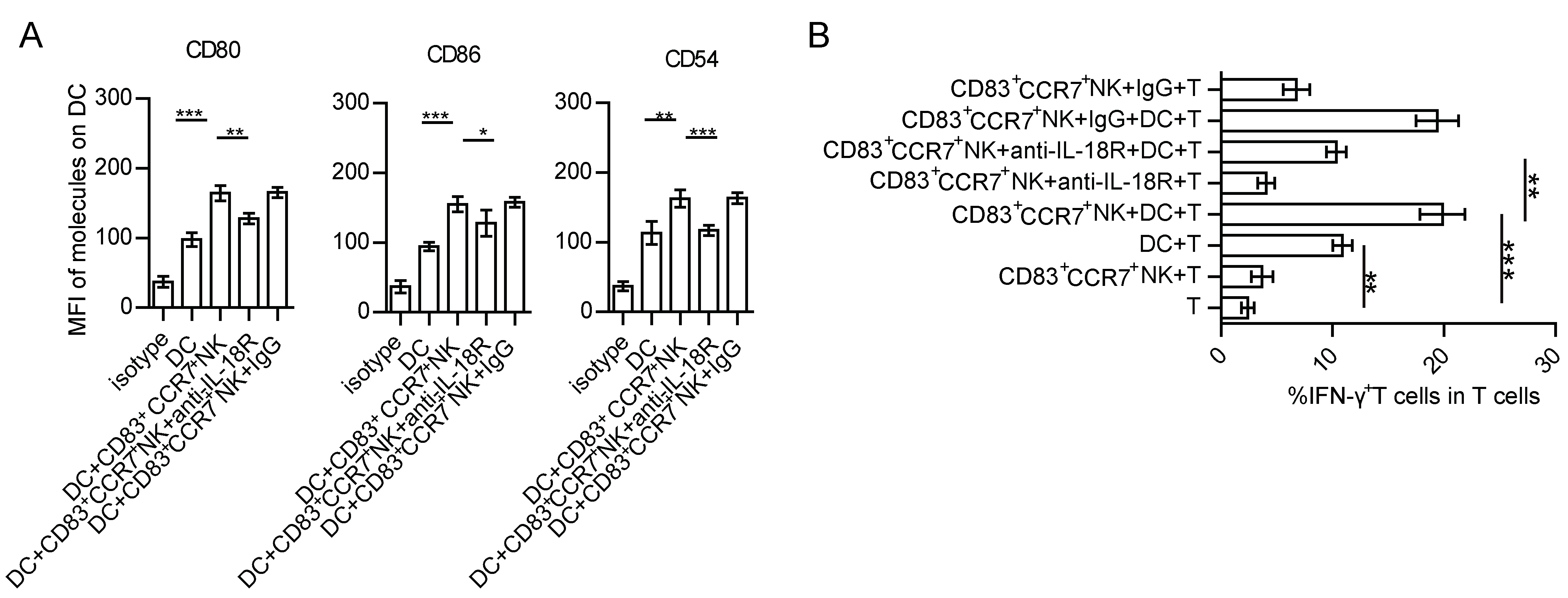

Supplement: Supplementary file 11 [file JCMM-23-1827-s011.tif]
