# Supplementary material for: CD83+CCR7+ NK cells induced by interleukin 18 by dendritic cells promote experimental autoimmune uveitis
Source: J Cell Mol Med. 2018 Dec 8;23(3):1827–39. doi: 10.1111/jcmm.14081 (PMC6378215; doi:10.1111/jcmm.14081)
Supplement: Supplementary file 12 [file JCMM-23-1827-s012.pdf]

CD83^{+}CCR7^{+} NK cells induced by interleukin 18 by dendritic cells promote experimental autoimmune uveitis

Qiang Fu[a]* #, Xuejing Man[b]*, Xin Wang[c]*, Nannan Song[d], Yuanbin Li[b], Jiangnan Xue[a], Yufei Sun[a], Wei Lin[d]#

[a] Department of Immunology, Binzhou Medical University, Yantai, China 264003

[b]Department of Ophthalmology, Yuhuangding Hospital, Yantai, China 264001

[c]Department of Clinical Laboratory, Qilu Hospital, Shandong University, Jinan, China 250012

[d]Institute of Basic medicine, Shandong Academy of medical Sciences, Jinan, China 250062

*co-first author,

#Correspondence to Qiang Fu, Email: qiangfu11@fudan.edu.cn or Wei Lin, Email: weilin11@fudan.edu.cn

Supplementary Figure and Figure legends

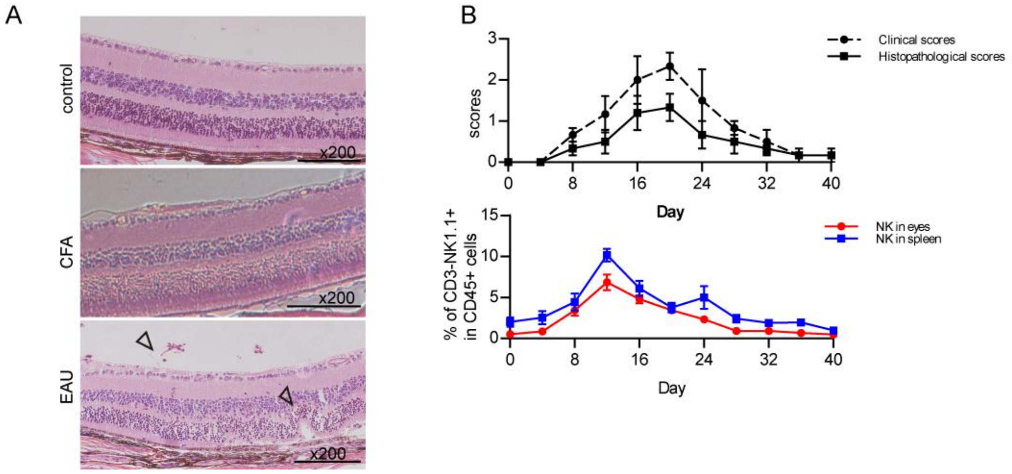

Supplementary Figure 1. The histopathology change of EAU mice. **(A)** Histopathology of a representative section from the eye of a control (day 0), an immunized mouse by $IRBP_{1-20}$ and PTX on day 16 and an immunized mouse by CFA (hematoxylin and eosin, original magnification, ×200). Infiltrating lymphocytes, vasculitis and photoreceptor folding within the retina were present within the inflamed eye (hollow arrows). **(B)** Relationship between histopathological and/or clinical scores of uveitis disease (upper panel) and the percentage of $CD3^{-}NK1.1^{+}$ cells in eyes (red line) or spleen (blue line) of EAU mice (bottom panel, fifteen mice were sampled at each time point and experiments were independently replicated three times).

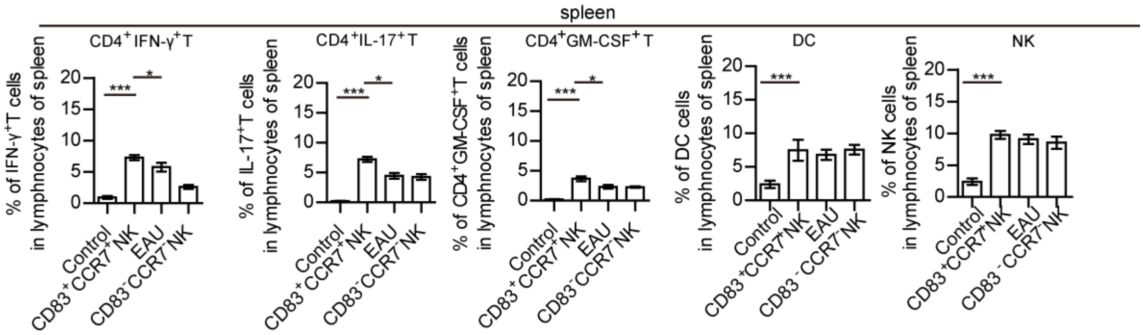

Supplementary Figure 2. Percentage of CD4$^+$ IFN-γ$^+$ T cells, CD4$^+$ IL-17$^+$ T cells, CD4$^+$ GM-CSF$^+$ T cells, CD11b$^+$CD11c$^+$MHC-II$^+$ DCs and CD3$^-$NK1.1$^+$ NK cells in the spleen of mice receiving CD83$^+$CCR7$^+$ NK or CD83$^-$CCR7$^-$ NK cell transfer as compared with that of EAU mice (N=5/group and the experiment was replicated three times, values represent the mean ± s.e.m., ANOVAs test, ****P*<0.001,**P*<0.05).

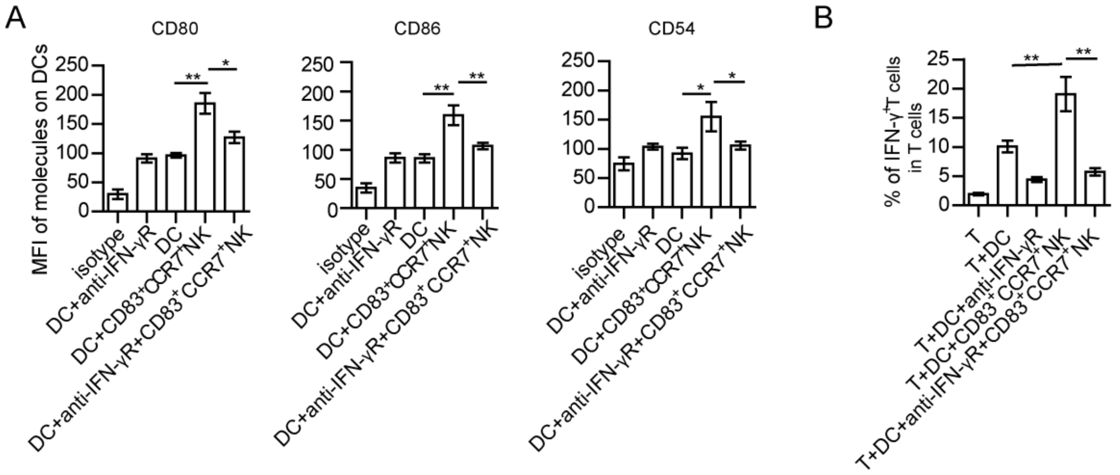

Supplementary Figure 3. With CD83$^+$CCR7$^+$ NK cells and/or anti-IFN-γR antibody treatment, the maturation of DCs is detected. **(A)** With or without anti-IFN-γR antibody treatment, the expression of CD80, CD86, CD54 within single DCs or these co-cultured with CD83$^+$CCR7$^+$NK cells. Mean fluorescence value (MFI) of CD80, CD86, and CD54 on DCs which were treated by CD83$^+$CCR7$^+$ NK cells, anti-IFN-γR antibody, anti-IFN-γR antibody and CD83$^+$CCR7$^+$ NK cells, or not were measured by flow cytometry. **(B)** With or without anti-IFN-γR antibody treatment, the percentage of IFN-γ$^+$ T cells in T cells or T cells cocultured with DCs, CD83$^+$CCR7$^+$NK cells pretreated-DCs, anti-IFN-γR antibody pretreated-DCs or anti-IFN-γR antibody pretreated-DCs co-cultured with CD83$^+$CCR7$^+$NK cells (A-B, N=10, values represent the mean±s.e.m., ANOVAs test, *P<0.05, **P<0.01,***P<0.001).

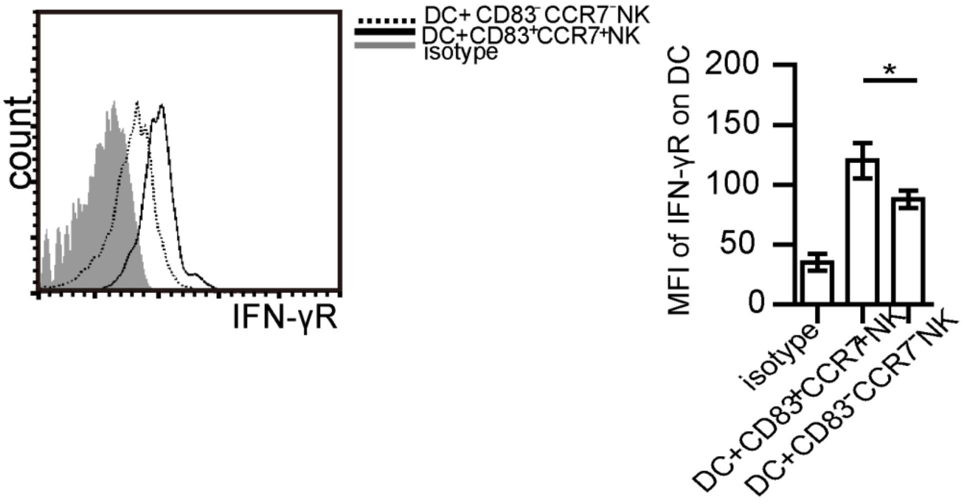

Supplementary Figure 4. The expression of IFN-γR on DCs co-cultured with CD83$^+$CCR7$^+$NK cells or CD83$^-$CCR7$^-$NK cells. Mean fluorescence value (MFI) of IFN-γR on DCs co-cultured with CD83$^+$CCR7$^+$NK cells or CD83$^-$CCR7$^-$NK cells were measured by flow cytometry. (N=10, values represent the mean±s.e.m., ANOVAs test, *P<0.05).

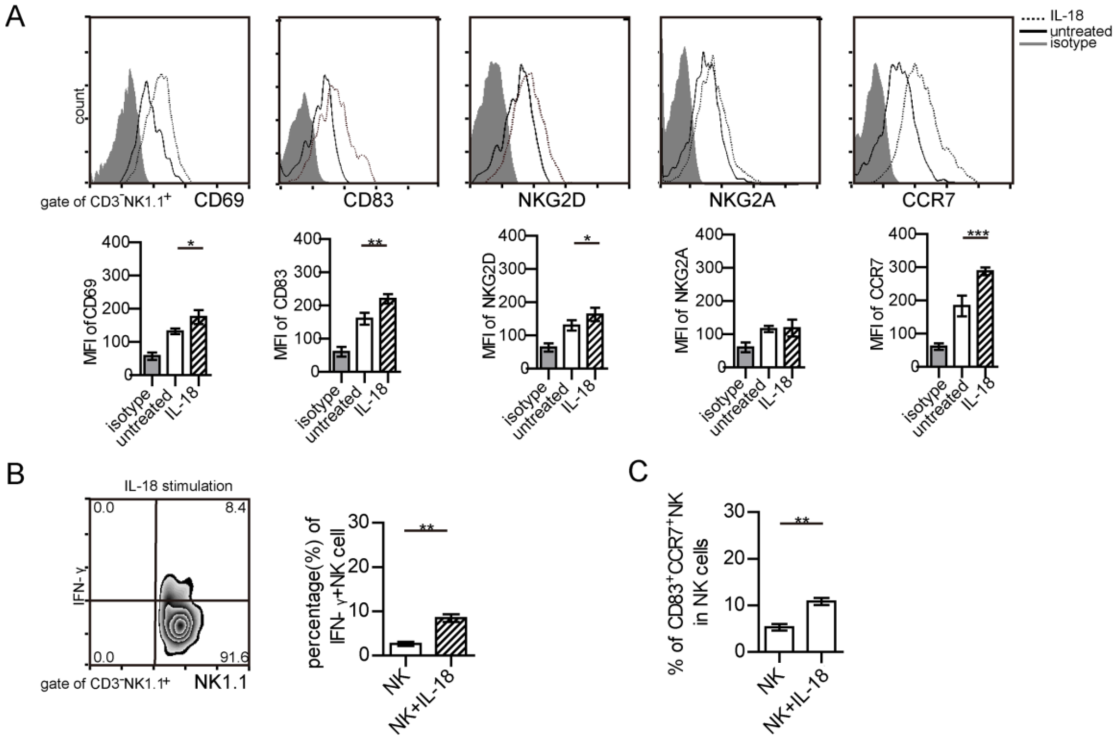

Supplementary Figure 5. Effect of IL-18 on NK cells in vitro. **(A)** Effect of IL-18 on the expression of CD69, CD83, NKG2D, NKG2A and CCR7 within NK cells and **(B)** IFN-γ production in NK cells (N=15, values represent the mean±s.e.m. Two-tailed Student's t-tests, **P*<0.05, ***P*<0.01,****P*<0.001). **(C)** With IL-18 inducing, the percentage of $CD83^{+}CCR7^{+}$NK cells in NK cells (N=15, values represent the mean±s.e.m. Two-tailed Student's t-tests, ***P*<0.01).

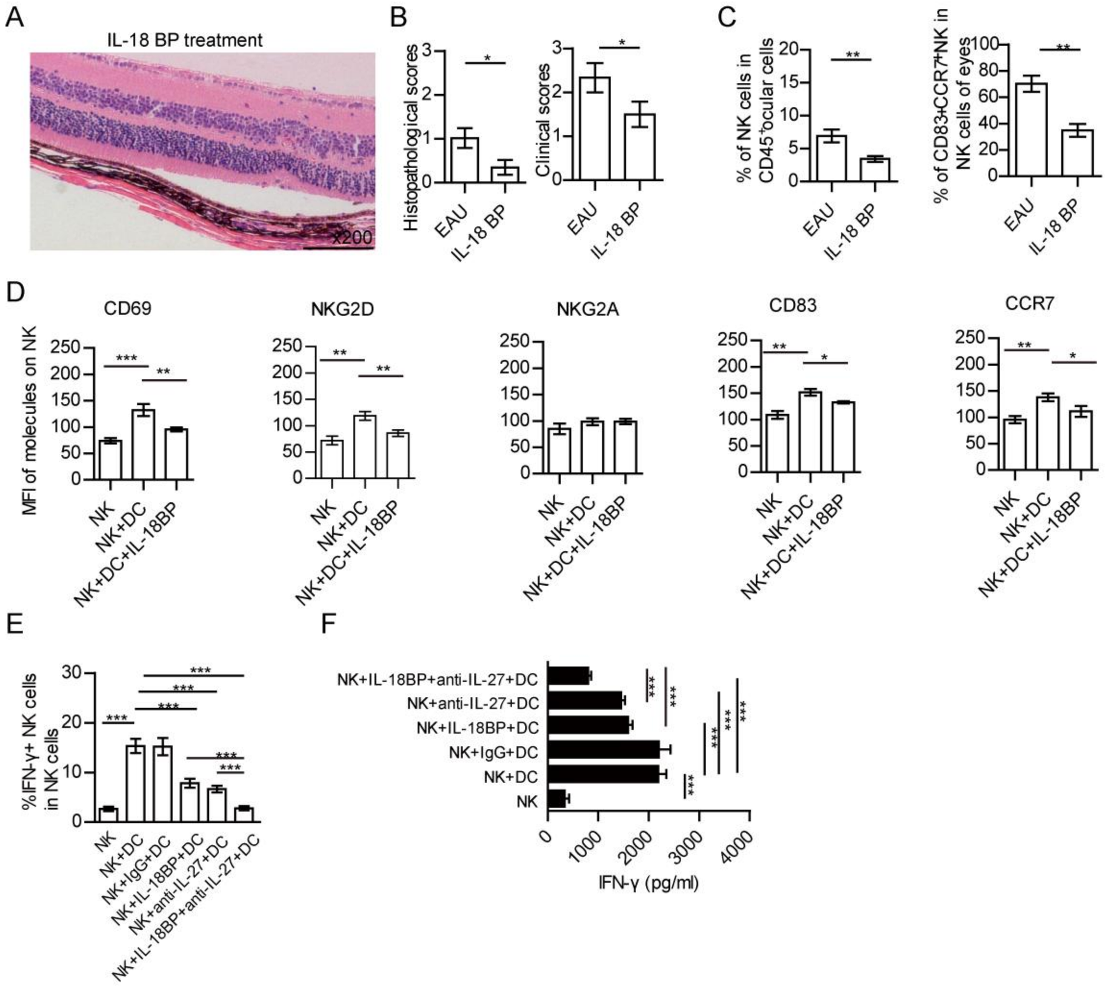

Supplementary Figure 6. IL-18BP treatment decreased the symptom and the number of CD83$^+$CCR7$^+$NK cells of eyes of EAU, and decreased the effect of DCs on the activation of NK cells in vitro. **(A)** Histopathology of a representative section from the eyes of IL-18 BP treated EAU mice. **(B)** The histopathological and/or clinical scores of IL-18 BP treated EAU mice, compared with untreated EAU mice. **(C)** The percentage of NK cells or CD83$^+$CCR7$^+$NK cells in the eyes of IL-18 BP treated EAU mice or untreated EAU mice. (B-C: N=10, values represent the mean±s.e.m., Two-tailed Student's t-tests, **P*<0.05, ***P*<0.01). **(D)** In vitro experiment, the expression of CD69, NKG2D, NKG2A, CD83 and CCR7 in NK cell, which were cocultured with DC with IL-18BP treated or not. Mean fluorescence value (MFI) of CD69, NKG2D, NKG2A, CD83 and CCR7 in NK cell cocultured with IL-18BP pretreated DC, compared with these on NK cell cocultured with DC, or NK cell. **(E)** With IL-18BP, anti-IL-27 neutralizing antibody, or both of them treatment in vitro,

percentage of IFN-γ$^+$NK cells in NK cells cocultured with DCs, compared with these without IL-18BP treating. (**F**) With IL-18BP, anti-IL-27 neutralizing antibody, or both of them treatment in vitro, the concentration of IFN-γ in the co-culture of DC and NK were measured to compare with these without treatment. (**D-F**): the experiment was replicated three times, values represent the mean±s.e.m., ANOVAs test, *P<0.05, **P<0.01, ***P<0.001).

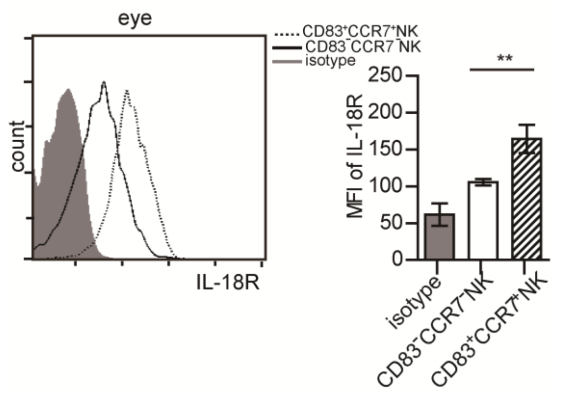

Supplementary Figure 7. The expression of IL-18R within $CD83^{+}CCR7^{+}$NK, or $CD83^{-}CCR7^{-}$NK cells. Mean fluorescence value (MFI) of IL-18R on $CD83^{+}CCR7^{+}$NK cells were higher than these on $CD83^{-}CCR7^{-}$NK cells (N=10, values represent the mean ± s.e.m., ANOVAs test, *P<0.05, **P<0.01,***P<0.001).

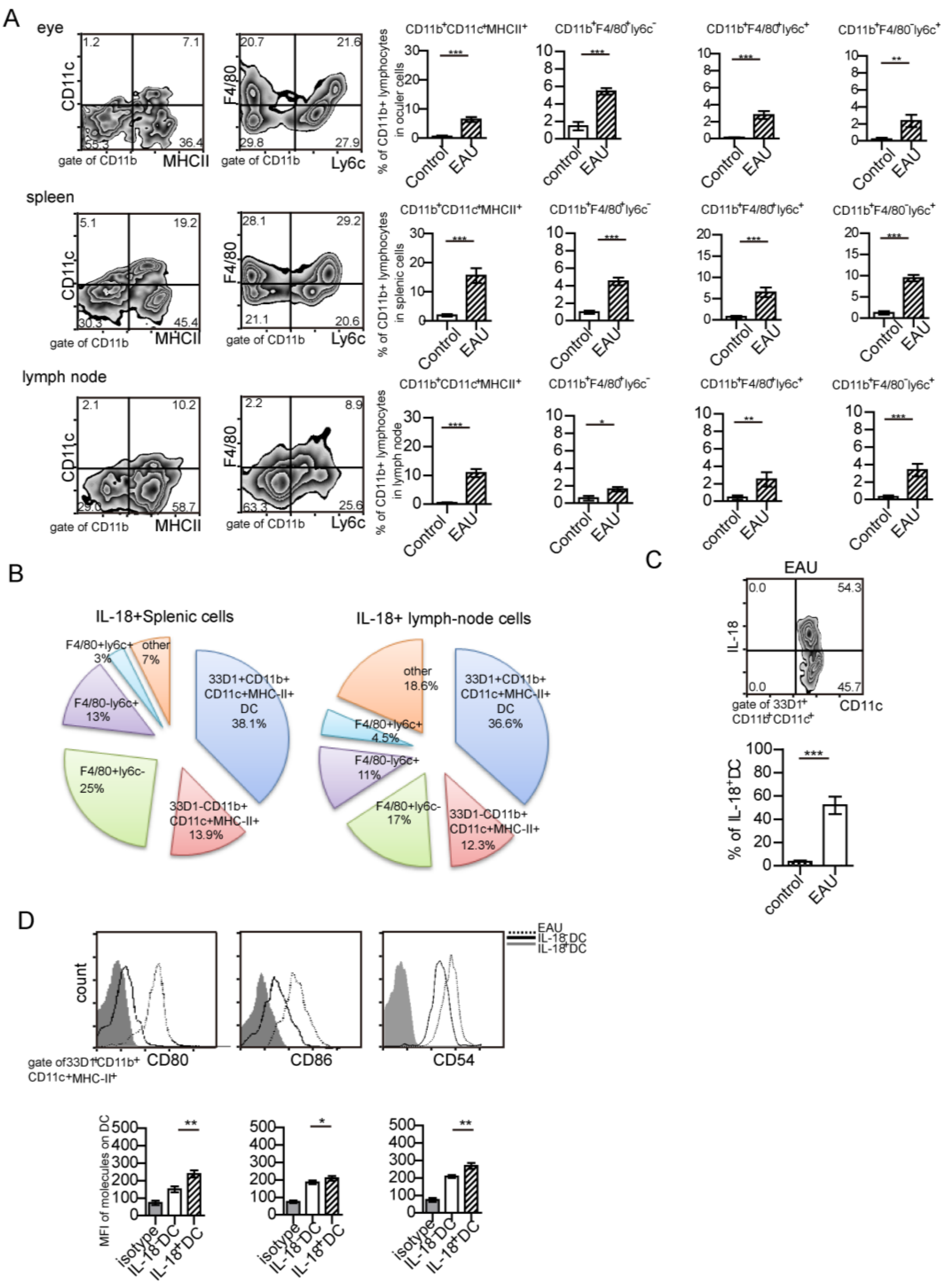

Supplementary Figure 8. IL-18 positive subset cells in increased CD11b positive cells of EAU. **(A)** Increased CD11b positive subset cells in the eyes, lymph nodes and spleen of EAU as compared with that from control mice (Data were from three independent experiments, N=30, values represent the mean±s.e.m., Two-tailed Student's t-tests,**P*<0.05,***P*<0.01,****P*<0.001). (**B)** The proportion of cell subsets in IL-18 positive cells from spleen or lymph nodes of EAU. **(C)** The percent of IL-18$^+$ DCs in DCs of EAU, compared with these of control mice. (Ten mice were used for

each group and the experiment was replicated three times, values represent the mean ± s.e.m., Two-tailed Student's t-tests, ****P*<0.001). **(D)** Expressions of CD80, CD86 and CD54 within IL-18^{+} DCs of EAU as compared with that IL-18^{-} DCs. (The experiment was replicated three times, values represent the mean ± s.e.m., ANOVAs test, **P*<0.05,***P*<0.01).

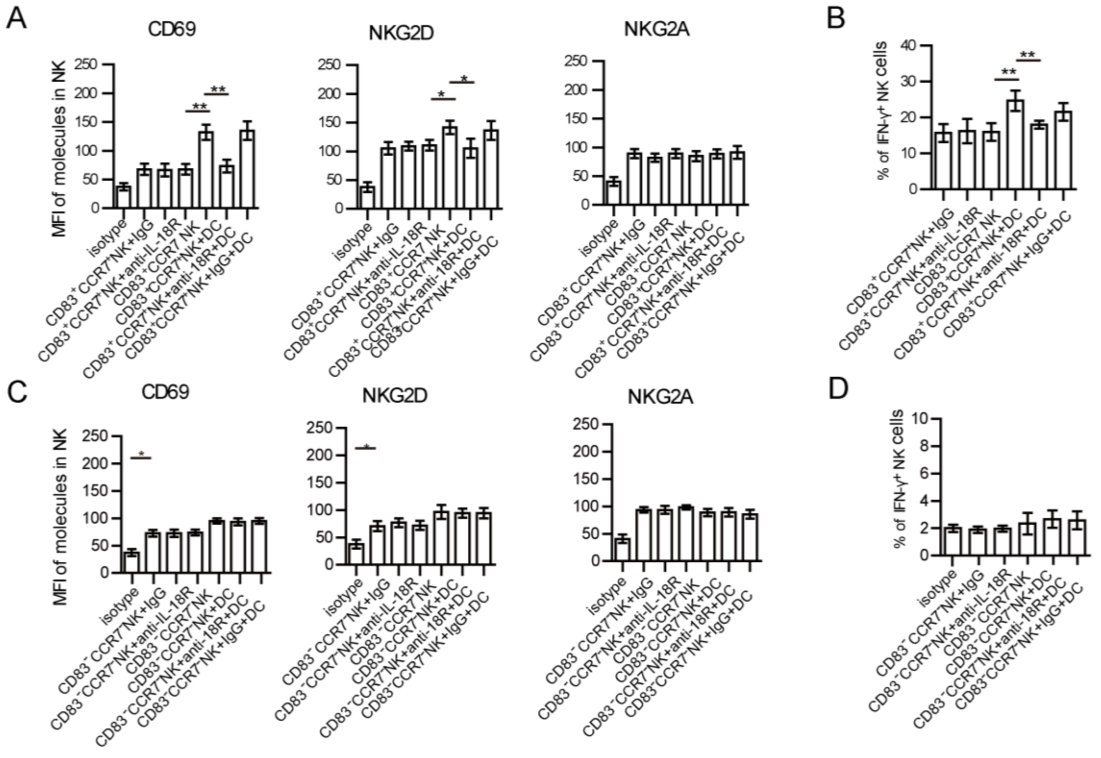

Supplementary Figure 9. Blocking IL-18R on CD83$^+$CCR7$^+$NK cells or CD83$^-$CCR7$^-$NK cells, the effect of isolated 33D1$^+$CD11b$^+$CC11c$^+$MHC-II$^+$ DCs on the activation of NK cells were determined. **(A)** The expressions of CD69, NKG2D, and NKG2A in CD83$^+$CCR7$^+$NK cells or NK cells co-cultured with DCs, or anti-IL-18R-pretreated-NK cells co-cultured with DCs, IgG-pretreated-NK cells co-cultured with DCs. **(B)** The secretion of IFN-γ in CD83$^+$CCR7$^+$NK cells with or without DCs co-culture or anti-IL-18R-pretreated-NK cells co-cultured with DCs, IgG- pretreated-NK cells co-cultured with DCs were measured. **(C)** The expressions of CD69, CD83, NKG2D, NKG2A and CCR7 in CD83$^-$CCR7$^-$NK cells or NK cells co-cultured with DCs, or anti-IL-18R-pretreated-CD83$^-$CCR7$^-$NK co-cultured with DCs, IgG-pretreated-NK cells co-cultured with DCs. **(D)** The secretion of IFN-γ in CD83$^-$CCR7$^-$NK cells with or without DCs co-culture or anti-IL-18R-pretreated-NK cells co-cultured with DCs, IgG-pretreated-NK cells co-cultured with DCs were measured (A-D: N=10, values represent the mean±s.e.m., ANOVAs test, *P<0.05, **P<0.01).

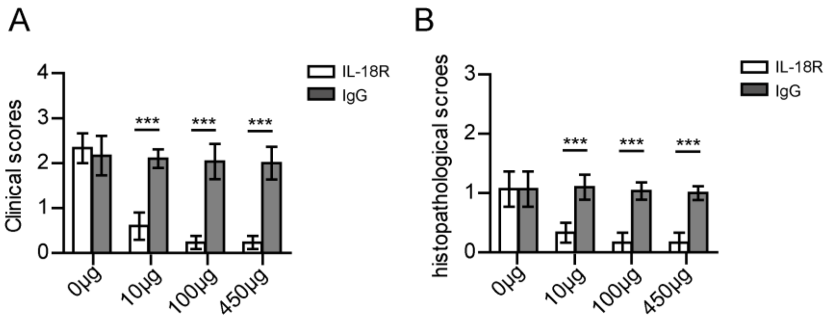

Supplementary Figure 10. With different doses of anti-IL-18R treatment, the clinical and histological scores of EAU were measured. With different doses of anti-IL-18R treatment, the clinical (A) and histological scores (B) of EAU were evaluated after treatment 8 days (N=3/group, values represent the mean±s.e.m., ANOVAs test, **P*<0.05, ***P*<0.01,****P*<0.001).

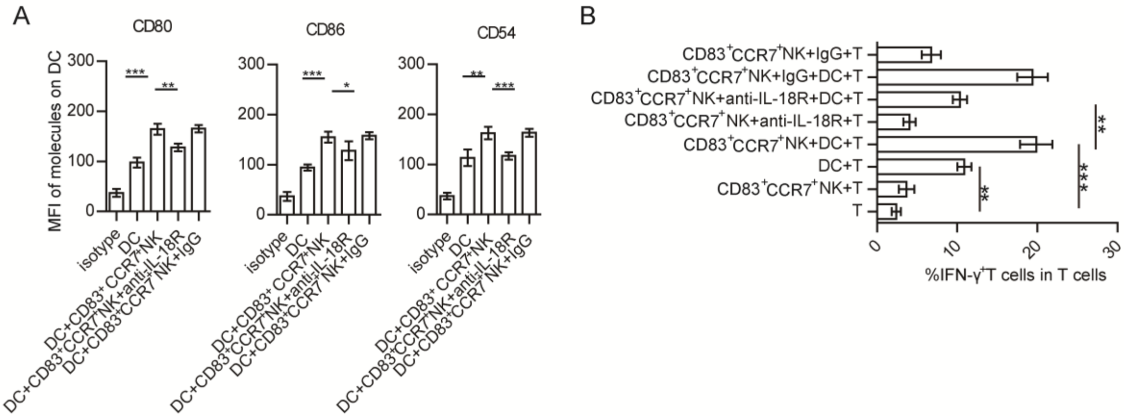

Supplementary Figure 11. Treating NK cells or blocking IL-18R on CD83$^+$CCR7$^+$NK cells, the activation of DCs and IFN-γ secretion in T cells which cocultured with DCs were decreased. **(A)** The expressions of CD80, CD86 and CD54 in DCs co-cultured with NK, or anti-IL-18R-pretreated-NK cells, or IgG-pretreated-NK cells. **(B)** The secretion of IFN-γ in T cells, which were treated with CD83$^+$CCR7$^+$NK cells with or without DCs co-culture or anti-IL-18R-pretreated-NK cells co-cultured with DCs, IgG- pretreated-NK cells co-cultured with DCs, were measured (N=10, values represent the mean±s.e.m., ANOVAs test, **P*<0.05, ***P*<0.01,****P*<0.001).
